# Virtual Clinical Studies to Examine the Probability Distribution of the AUC at Target Tissues Using Physiologically-Based Pharmacokinetic Modeling: Application to Analyses of the Effect of Genetic Polymorphism of Enzymes and Transporters on Irinotecan Induced Side Effects

**DOI:** 10.1007/s11095-017-2153-z

**Published:** 2017-04-10

**Authors:** Kota Toshimoto, Atsuko Tomaru, Masakiyo Hosokawa, Yuichi Sugiyama

**Affiliations:** 1 Sugiyama Laboratory, RIKEN Innovation Center, RIKEN, 1-7-22 Suehiro-cho, Tsurumi-ku, Yokohama, Kanagawa 230-0045 Japan; 2 Faculty of Pharmaceutical Sciences, Chiba Institute of Science, Choshi, Japan

**Keywords:** Cluster Newton method, inter-individual variability, irinotecan(CPT-11), neutropenia, physiologically-based pharmacokinetic modeling, virtual clinical study

## Abstract

**Purpose:**

To establish a physiologically-based pharmacokinetic (PBPK) model for analyzing the factors associated with side effects of irinotecan by using a computer-based virtual clinical study (VCS) because many controversial associations between various genetic polymorphisms and side effects of irinotecan have been reported.

**Methods:**

To optimize biochemical parameters of irinotecan and its metabolites in the PBPK modeling, a Cluster Newton method was introduced. In the VCS, virtual patients were generated considering the inter-individual variability and genetic polymorphisms of enzymes and transporters.

**Results:**

Approximately 30 sets of parameters of the PBPK model gave good reproduction of the pharmacokinetics of irinotecan and its metabolites. Of these, 19 sets gave relatively good description of the effect of UGT1A1 *28 and SLCO1B1 c.521T>C polymorphism on the SN-38 plasma concentration, neutropenia, and diarrhea observed in clinical studies reported mainly by Teft *et al*. (Br J Cancer. 112(5):857-65, 20). VCS also indicated that the frequency of significant association of biliary index with diarrhea was higher than that of UGT1A1 *28 polymorphism.

**Conclusion:**

The VCS confirmed the importance of genetic polymorphisms of UGT1A1 *28 and SLCO1B1 c.521T>C in the irinotecan induced side effects. The VCS also indicated that biliary index is a better biomarker of diarrhea than UGT1A1 *28 polymorphism.

**Electronic supplementary material:**

The online version of this article (doi:10.1007/s11095-017-2153-z) contains supplementary material, which is available to authorized users.

## Introduction

**Fig. 1:**
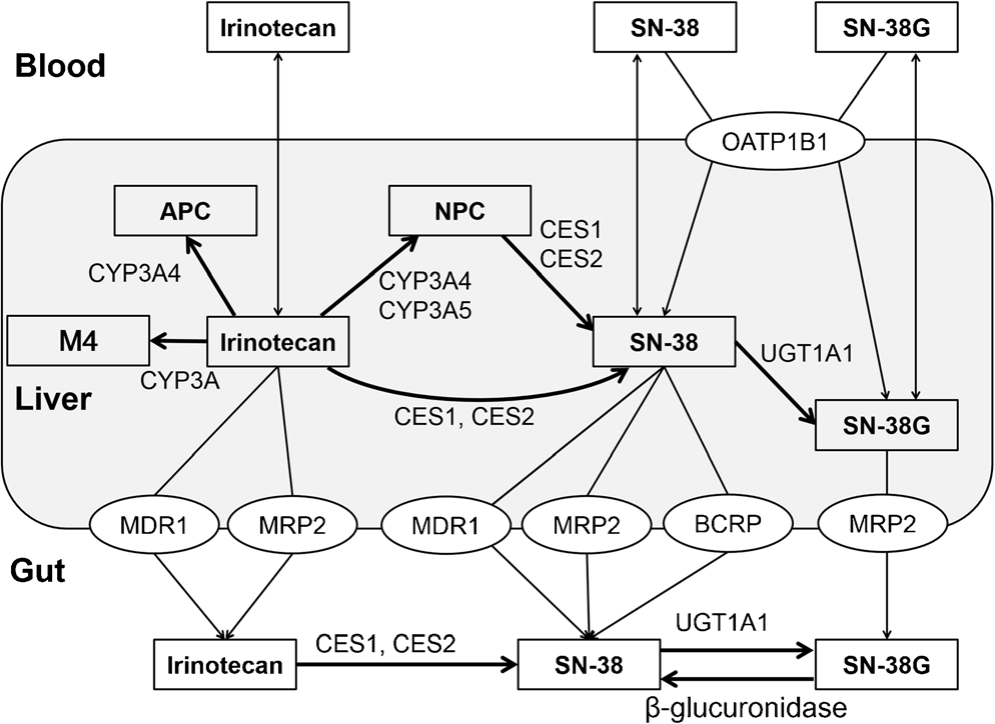
Metabolic and transport pathway for irinotecan (CPT-11).

Irinotecan (CPT-11) is a prodrug for the treatment of various kinds of cancer. Irinotecan exerts its pharmacological effect by blocking DNA replication and transcription by topoisomerase-1 inhibition (1). Irinotecan has a complex metabolic pathway in which carboxylesterase (CES) 1 and CES2 in the liver, gastrointestinal tract, and plasma convert irinotecan to its active metabolite SN-38 as a first step. SN-38 is then metabolized to the inactive metabolite SN-38 glucuronide (SN-38G) by uridine diphosphate glucuronosyltransferases (UGTs), especially UGT1A1. Deconjugation from SN-38G to SN-38 occurs by intestinal β-deglucuronidase. In addition, irinotecan undergoes CYP3A4/5 metabolism to inactive metabolites APC, M4, and NPC with further conversion to SN-38 by CES1 and CES2 (Fig. 1) (2).

The severe adverse effects of irinotecan are neutropenia and delayed diarrhea, which are caused by high exposure of plasma and intestinal epithelia to SN-38 (3). Irinotecan has been widely studied by clinical pharmacogenomics focused on UGT1A1 (4,5). A UGT1A1 *28 or UGT1A1 *6 phenotype may increase the risk of neutropenia owing to higher SN-38 exposure by decreasing the UGT1A1 metabolic clearance, as found in various clinical studies (6,7). In addition to the UGT1A1, some transporter genotypes are reported as having key roles in the adverse effects of Irinotecan. Decreased uptake clearance among patients with an SLCO1B1 (OATP1B1) c.521T>C genotype causes severe irinotecan toxicity (8). Although other clinical studies report that ABC transporter genotypes such as ABCB1 (MDR1), ABCG2 (BCRP), and ABCC2 (MRP2), which are involved in biliary excretion and intestinal efflux in the intestinal lumen, have been linked to exposure to SN-38 and the side effects of irinotecan (9,10), others have described that these genetic polymorphisms have no association with the adverse effects (11,12). Thus, different findings by various clinical studies have been made with regard to the associations between enzymes/transporters and drug-induced side effects (neutropenia, diarrhea). This is probably because insufficient numbers of patients were included in most of those previous clinical studies.

By contrast, to predict drug pharmacokinetics quantitatively, physiologically-based pharmacokinetic (PBPK) modeling has been more recently used to predict blood and tissue concentration–time profiles. Over 350 articles on human PBPK modeling have been published since 2008 (13). The number of regulatory submissions to the U.S. FDA using PBPK model analysis has gradually increased since 2008 (14). Furthermore, the potential of virtual clinical study (VCS), which uses computational simulation with PBPK, pharmacodynamic, and toxicodynamic models—with virtual patients generated by the variability of physiological and pharmacokinetic parameters based on genetic polymorphism, ethnic differences, and inter- and intra-individual variability—is an advantage of PBPK model analysis (15). The concept of VCS has already been integrated into PBPK model analysis, where the association between SLCO1B1 genotype and rosuvastatin pharmacological effect (a decline of the plasma concentration of mevalonic acid) has been reported (16). In VCS, since the values of all parameters for each virtual patient are known, it is possible to identify the factors significantly associated with the effects and side effects based on the large number of simulation results. It was considered that an accurate PBPK model of irinotecan and its metabolites would enable us to identify the factors related to its side effects. A brief PBPK model of irinotecan and SN-38 metabolism has been published by Fujita *et al*. (17). They predicted SN-38 exposure after renal failure. However, because such a simple PBPK model did not contain an enterocyte compartment, it was impossible to predict diarrhea.

The purpose of the present study was to gain insight into the factors highly associated with neutropenia and diarrhea using a VCS approach with the PBPK model. The parameters for the PBPK model, which could reproduce the average blood concentration–time profile of irinotecan and its metabolites, were estimated as the first step of our analyses. Because there were 46 unknown parameters within the search space, it was difficult to set appropriate initial values for unknown parameters; thus, conventional nonlinear least squares methods such as the Gauss–Newton method and Levenberg–Marquardt method could not be applied. To overcome this problem, a Cluster Newton method (CNM) was newly employed to optimize unknown parameters (18). In this algorithm, multiple sets of initial values of parameters were first generated at random from a certain range of initial values for each unknown parameter. Then, linear approximations of projection from parameters to objective function were used to determine the next iterations from initial sets. CNM has some advantages over conventional parameter optimization algorithms (19). The first advantage is that it is easy to set the initial condition of the parameters. While CNM only requires a rough range of values for each parameter, many conventional approaches require fixed initial possible values. The second advantage is that multiple optimized sets of parameters can be obtained using a single optimization.

In the present study, 46 unknown parameters in the PBPK model of the metabolism of irinotecan and its metabolites were optimized by CNM. Then, obtained sets of parameters were evaluated regarding whether the PBPK model with optimized parameters could reproduce the outcomes of clinical studies, focusing on the effect of UGT1A1 *28 and SLCO1B1 c.521T>C polymorphism, as reported by Teft *et al*. (20) and in some other clinical studies, and the reproduction of clinical outcomes was attempted using a VCS approach.

## Materials and Methods

### Measurement of the Contribution of CES1 Metabolism of Irinotecan to SN-38

The possible involvement of CES1 in the metabolism of irinotecan in the liver was assessed using anti-CES1A IgG. The methods were as follows.

CES1A IgG-treated microsomes were prepared by a modification of a procedure described previously (21). Microsomes (50 mixed-sex donors, Corning, Japan) were solubilized with 0.5% cholic acid in 10 mM Tris-HCl buffer (pH 8.0). After solubilizing, this mixture was centrifuged, and anti-CES1A IgG was added to the supernatant and incubated for 30 min at 37°C, and then for 24 h at 4°C. After the incubation, nProtein A Sepharose 4 Fast Flow (GE Healthcare, Japan) was added to the mixture, which was then incubated for 1 h on ice. Then, this mixture was centrifuged and the hydrolase activity in the supernatant was determined.

The method for assessment of hydrolase activity was modified from a procedure described previously (22). To assess hydrolase activity, temocapril was used as a positive control for CES1 activity. Temocapril (1 μM) and irinotecan (2 μM) were added to the CES1A IgG-treated or control microsomes (0.1 mg/mL) and incubated at 37°C for up to 30 min. After the incubation, the reaction was stopped by the addition of 0.1% formic acid in acetonitrile containing an internal standard. The generation of metabolites (SN-38 for irinotecan and temocaprilat for temocapril) was measured by LC-MS/MS. The LC-MS/MS consisted of a Nexera X2 separating module (Shimadzu, Kyoto, Japan) and an LCMS8050 mass spectrometer (Shimadzu) with an electron ion spray interface. The mass spectrometer was operated in a multiple reaction monitoring mode using the respective MH^+^ ions, m/z 587 to m/z 124 for irinotecan, m/z 393 to m/z 349 for SN-38, m/z 477 to m/z 270 for temocapril, m/z 449 to m/z 270 for temocaprilat, and m/z 277 to m/z 175 for chlorpropamide (internal standard). Chromatographic separation was performed on a C18 column (Kintex C18, 2.1 × 100 mm, 2.6 μm; Phenomenex Inc., Torrance, CA) at 40°C under gradient conditions (0.1% formic acid and acetonitrile) at 0.2 mL/min. The stabilities of SN-38 and temocaprilat were confirmed up to 20 h, and no degradation was observed during the procedure.

### Development of the PBPK Model of Irinotecan and Its Metabolites

**Fig. 2:**
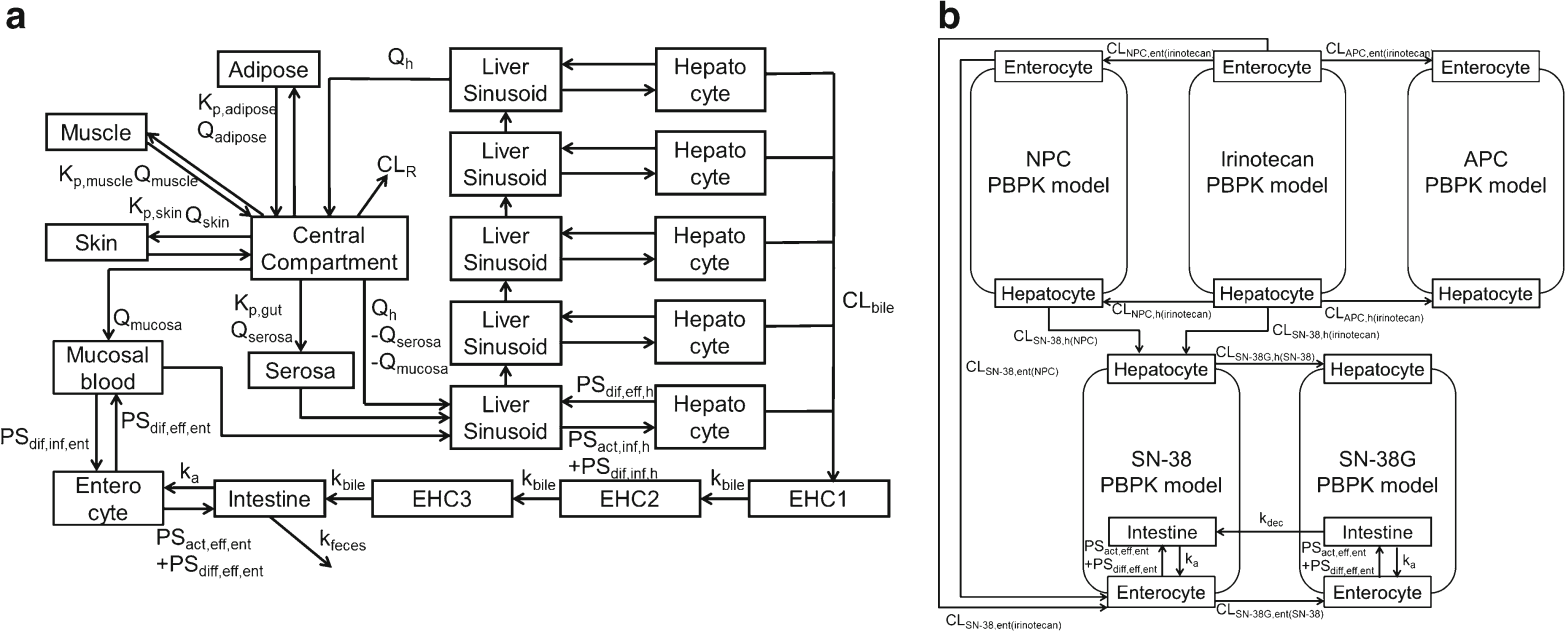
Structures of the physiologically-based pharmacokinetic (PBPK) model of irinotecan and its metabolites. (**a**) shows a module of PBPK model. The liver compartment was adapted as a permeability-limited model and divided into five compartments (23) to construct a model similar to the dispersion model. Enterohepatic circulation was considered. Permeability between the intestinal lumen and enterocytes and between enterocytes and mucosal blood was also incorporated. Portal vein blood flow was divided into mucosal blood flow (Q_mucosa_) and serosal blood flow (Q_serosa_). (**b**) Each module for irinotecan and the metabolites is connected shown here based on the scheme in Fig. 1.

The PBPK model of irinotecan and its metabolites SN-38, SN-38G, NPC, and APC is shown in Fig. 2. A module of the PBPK model shown in Fig. 2a was developed and connected as depicted in Fig. 2b. For each module, the liver compartment was divided into five units of extrahepatic and hepatocyte compartments to mimic a dispersion model (5 liver model (23)) and take the permeability-limited process of hepatic uptake into account. As biliary excretion of irinotecan and its metabolites was observed from a mass balance study of a patient with a biliary T-tube (24), three transit compartments between hepatocytes and the intestinal lumen were incorporated to represent enterohepatic circulation. The structure of a segregated flow model (25) that could represent intestinal metabolism and transport was employed.

Fixed physiological and physicochemical parameters were obtained from the literature and our experiments (Supplementary Table 1). Tissue-to-blood concentration ratios (K_p,muscle_, K_p,skin_, K_p,adipose_, and K_p,gut_), and protein unbound fraction in the liver (f_h_) and enterocytes (f_gut_) were based on reported in silico calculations using clogP and pKa (acidic and basic) obtained from SciFinder Scholar (Chemical Abstracts Service, Columbus, OH) (26,27). The name and initial range of unknown parameters for CNM optimization are described in Table I. In the liver compartment, parameter optimization with some hybrid parameters (CL_int,all_, R_dif,h_, β, f_bile_) (28) was performed, except for irinotecan. PS_act,inf,h_ was defined as the hepatic active uptake via transporters, PS_dif,inf,h_ as the influx clearance by passive diffusion through sinusoidal membrane in the liver, PS_dif,eff,h_ as the efflux clearance by passive diffusion through the sinusoidal membrane in the liver, CL_met,h_ as the metabolic clearance in the liver, and CL_bile_ as the biliary excretion clearance. Then, the overall intrinsic clearance (CL_int,all_) was described in Eq. 1 (29).

1 $$ { C L}_{int, all}=\left({ P S}_{act, inf, h}+{ P S}_{dif, inf, h}\right)\cdot \frac{C{ L}_{met, h}+{ C L}_{bile}}{P{ S}_{dif, eff, h}+{ C L}_{met, h}+{ C L}_{int, bile}} $$

**Table I:** Initial Range and Theoretical Lower Limit of Parameters for CNM

A. Common parameters							
Parameter	Unit	Irinotecan	SN-38	SN-38G	NPC	APC	Theoretical lower limit
V_central_	L/kg	0.075 ~ 0.75	0.075 ~ 0.75	0.075 ~ 0.75	0.075 ~ 0.75	0.075 ~ 0.75	0.074^a^
k_a_	/h	0.06 ~ 6.0	0.06 ~ 6.0	0.06 ~ 6.0	0.06 ~ 6.0	0.06 ~ 6.0	0
k_feces_	/h	0.06 ~ 6.0	0.06 ~ 6.0	0.06 ~ 6.0	0.06 ~ 6.0	0.06 ~ 6.0	0
R_dif,h_	-	-^b,c^	0.01 ~ 1.0	0.01 ~ 1.0	-^c^	-^c^	0
1/β	-	-^b^	1.1 ~ 10	1.1 ~ 10	1.1 ~ 10	1.1 ~ 10	1
CL_int,all_	L/h/kg	-^b^	0.1 ~ 100	0.1 ~ 100	0.1 ~ 100	0.1 ~ 100	0
1/f_bile_	-	-^b^	-^e^	-^d^	1.1 ~ 10	-^d^	1
k_bile_	/h	0.06 ~ 6.0	0.06 ~ 6.0	0.06 ~ 6.0	0.06 ~ 6.0	0.06 ~ 6.0	0
R_dif,ent_	-	0.1 ~ 10	0.1 ~ 10	0.1 ~ 10	-^c^	-^c^	0
B. Irinotecan parameters							
Parameter	Unit	Value	Theoretical lower limit				
PS_dif,inf,h_	L/kg	0.1 ~ 100	0				
CL_SN-38,h(irinotecan)_	L/h/kg	0.1 ~ 100	0				
CL_NPC,h(irinotecan)_	L/h/kg	0.1 ~ 100	0				
CL_APC,h(irinotecan)_	L/h/kg	0.1 ~ 100	0				
CL_others,h(irinotecan)_	L/h/kg	0.1 ~ 100	0				
CL_bile_	L/h/kg	0.1 ~ 100	0				
PS_dif,eff,ent_	L/h/kg	0.1 ~ 100	0				
CL_SN-38,h(irinotecan)_ /CL_SN-38,ent(irinotecan)_	-	1.1 ~ 10	1				
CL_NPC,h(irinotecan)_ / CL_NPC,ent(irinotecan)_	-	1.1 ~ 10	1				
C. SN-38 parameters							
Parameter	Unit	Value	Theoretical lower limit				
1/f_glu_^e^	-	1.1 ~ 10	1				
CL_SN-38G,h(SN-38)_ /CL_SN-38G,ent(SN-38)_	-	1.1 ~ 10	1				
D. SN-38G parameters							
Parameter	Unit	Value	Theoretical lower limit				
k_dec_	/h	0.06 ~ 6	0				

^a^V_central_ is larger than blood volume

^b^Hybrid parameters were not used. Instead of it, as shown in Table 1B. parameters for representing elementary steps were used

^c^No involvement of active uptake or efflux transporters for these compounds

^d^f_bile_ value was fixed as 1

^e^The ratio (f_glu_) of metabolic clearance for glucuronidation of SN-38 to hepatic intrinsic clearance was estimated using the following equation, f_glu_ = 1 - f_blie_

In the present research, PS_dif,inf,h_ = PS_dif,eff,h_ was assumed. R_dif,h_, β, and f_bile_ were defined as described in Eqs. 2, 3, and 4.

2 $$ {R}_{dif, h}=\frac{P{ S}_{dif, inf, h}}{P{ S}_{act, inf, h}} $$

3 $$ \upbeta =\frac{C{ L}_{met, h}+{ C L}_{int, bile}}{P{ S}_{dif, eff, h}+{ C L}_{met, h}+{ C L}_{int, bile}} $$

4 $$ {f}_{bile}=\frac{CL_{bile}}{CL_{met, h}+{CL}_{bile}} $$

As shown in Fig. 1, SN-38 is considered to be taken up by OATP1B1 into the liver based on a previous report (30). The uptake study of SN-38G was performed using an HEK293/OATP1B1-expressing cell system. The amount of SN-38G in the cell was measured by LC-MS/MS which was used the measurement of the contribution of CES1 metabolism of irinotecan to SN-38. The uptake of SN-38G by HEK293/OATP1B1 cells was showed time-dependent increase and higher than that by HEK293/mock cells (Supplementary Fig. 1). Therefore, SN-38G is also considered with OATP1B1-mediated uptake in the liver. No involvement of active uptake transporters for other compounds was assumed.

In the intestinal compartment, the value of PS_dif,eff,ent_, which represents efflux clearance by passive diffusion through a basolateral membrane from the enterocyte to the mucosal blood, and PS_dif,inf,ent_, which represents influx clearance by passive diffusion through basolateral membranes from the mucosal blood to the enterocyte, was assumed to be equal. The value of passive diffusional clearance from the enterocyte to the intestinal lumen was defined as the product of PS_dif,eff,ent_ and an apical/basolateral area ratio (AR = 20 (31)). PS_act,eff,ent_ represents the active efflux transporters via ABCB1, ABCG2, and ABCC2 in the intestine. A hybrid parameter R_dif,ent_ was defined as the ratio of passive diffusional efflux to the active efflux in the enterocytes as described in Eq. 5 for compounds that are substrates of these efflux transporters.

5 $$ {R}_{dif, ent}=\frac{AR^{\ast }\ {PS}_{dif, eff, ent}}{PS_{act, eff, ent}} $$

The differential equations of the PBPK model are described in the Supplementary Text.

The process employed to optimize parameters using CNM was as follows.

1. 10,000 initial sets of parameters, the range of which is described in Table I for each unknown parameter, were generated.
2. CNM was used to optimize the 10,000 sets of parameters. To provide the diversity of the problem solutions (optimized sets of parameters), AUC for each compound was used as the objective function for optimization. Only stage 1 of the CNM optimization (18) was performed to avoid overfitting and save computational time.
3. Procedures 1 and 2 described above were repeated 100 times with 10,000 different initial sets of parameters.
4. The blood concentration–time profile of irinotecan and its metabolites was simulated using 1,000,000 optimized sets of parameters. The weighted sum of squares (WSS) between the simulated and observed blood concentration–time profile described in Eq. 6 was calculated

6 $$ \mathrm{WSS}={\sum}_{i=1}^n\frac{{\left({y}_i-{y}_i^{\prime}\right)}^2}{y_i^2},\ {y}_i: i\mathrm{th}\kern0.20em \mathrm{observed}\ \mathrm{value},{y}_i^{\prime }: i\mathrm{th}\ \mathrm{predicted}\ \mathrm{value} $$

5. The top 30 sets of parameters that minimized the WSS between simulated and observed concentration–time profiles at the sampling points were selected.

The clinical study reported by van der Bol *et al*. (32) was used for observed AUC and blood concentration–time profiles of irinotecan, SN-38, SN-38G, NPC, and APC. 30 selected sets of parameters were used for VCS as described below and given in ascending order of WSS for IDs 1 to 30.

### Generation of Virtual Patients

To perform VCS, consideration of genetic polymorphisms of some enzymes and transporters is required, in addition to the inter-individual variability for each parameter of the PBPK model. This VCS considered 6 genetic polymorphisms of enzyme and transporters (UGT1A1*28, SLCO1B1 c.521T>C and c.388A>G, ABCG2 c.421C>A, ABCB1 c.3435C>T, and ABCC2 c.-24C>T). The contribution of each enzyme and transporter in the metabolism and transporter process is assumed as follows: UGT1A1, metabolism from SN-38 to SN-38G in the liver (CL_SN-38G,h(SN-38)_) and intestine (CL_SN-38G,ent(SN-38)_), contributions for both are 100%; SLCO1B1, hepatic active uptake of SN-38 and SN-38G (PS_act,inf,h(SN-38)_ and PS_act,inf,h(SN-38G)_), contributions for both are 100%; ABCG2, biliary excretion of SN-38 (CL_bile(SN-38)_) and active efflux from enterocytes to the intestinal lumen of SN-38 (PS_act,eff,ent(SN-38)_), contributions for both are 33%; ABCB1, biliary excretion of irinotecan and SN-38 (CL_bile(irinotecan)_ and CL_bile(SN-38)_) and active efflux of irinotecan and SN-38 from enterocytes to the intestinal lumen (PS_act,eff,ent(irinotecan)_ and PS_act,eff,ent(SN-38)_), contributions are 50% for irinotecan and 33% for SN-38; and ABCC2, biliary excretion of irinotecan, SN-38, and SN-38G (CL_bile(irinotecan)_, CL_bile(SN-38)_, and CL_bile(SN-38G)_) and active efflux of irinotecan, SN-38, and SN-38G from enterocytes to the intestinal lumen (PS_act,eff,ent(irinotecan)_, PS_act,eff,ent(SN-38)_, and PS_act,eff,ent(SN-38G)_), contributions are 50% for irinotecan, 33% for SN-38, and 100% for SN-38G. Supplementary Table 2 shows the activity ratio and the allele frequency for each genetic polymorphism obtained from experiments *in vitro* and *in vivo*.

A virtual patient was generated by performing Monte Carlo simulation following a given frequency distribution (average, coefficient of variation [CV], and the shape of distribution) for each parameter. Hybrid parameters (e.g. CL_int,all_, β, R_dif,h_, f_bile_, R_dif,ent_) used for CNM optimization were converted to each parameter representing elementary processes. The averages of physiological parameters are presented in Supplementary Table 3A. Other parameters were obtained by optimization using the CNM, and the average values were used together with their variabilities. The variabilities (CV) and the shape of distribution are described in Supplementary Table 3B. Because the observed blood concentration–time profile of irinotecan and its metabolites (32) used by the CNM was obtained from patients recruiting the same number of UGT1A1 *1/*1 and *1/*28 patients, the value of metabolic clearance of SN-38 to SN-38G (CL_SN-38G,h(SN-38)_ and CL_SN-38G,ent(SN-38)_) was recalculated for UGT1A1 *1/*1 using values obtained from the CNM as an average value between UGT1A1 *1/*1 and *1/*28.

The variability of each variable in performing Monte Carlo simulation was set as follows.

1. **Body weight and body surface area**

The body weight of a virtual patient was generated based on normal distribution (average and CV are given in Supplementary Table 3). Body surface area (BSA) was obtained from a normal distribution of which the average and CV were calculated from Eq. 7 (33) and 5.5%, respectively.

7 $$ BSA=4.688\cdot {BW}^{\left(0.8168-0.0154\cdot \log (BW)\right)},\kern2em  BSA\left[{cm}^2\right], BW\left[ g\right] $$

CV of 5.5% was determined as follows. After temporary BSA was calculated from Eq. 7 with the distribution of body weight for a healthy man (average, 78.8 kg; CV, 11.7% (34)), actual BSA was calculated for the normal distribution of variability based on a certain set CV. Calculated BSA distribution was compared with the reported distribution (average, 1.97 m^2^; CV, 9.6% (35)). CVs estimated using the calculated and reported BSA distribution corresponded with each other. In this VCS, the average and CV of body weight distribution were also estimated to compare the calculated distribution of BSA with the distribution reported after clinical study of irinotecan (5,32).

(2) **Plasma and blood unbound fraction of compounds**

Plasma unbound fraction of compounds was calculated from Eq. 8, which was derived from the Langmuir equation.

8 $$ {f}_p=\frac{1}{1+\frac{n\left[ Pt\right]}{K_d}} $$

In Eq. 8, *n* represents the number of binding sites of the protein, [Pt] the protein concentration, and K_d_ the dissociation constant. f_p_ for each virtual patient was calculated so that *n*[Pt]/K_d_ was generated from the distribution described in Supplementary Table 3. Blood unbound fraction was calculated from Eq. 9 assuming the same unbound concentration of the compound between the plasma and blood cells:

9 $$ {f}_b=\frac{f_p}{1-\left(1-\frac{f_p}{f{}_r}\right) Hct} $$

where f_r_ represents the unbound fraction in the blood cells and Hct the hematocrit. Eq. 8 can be used to calculate f_r_. In these calculations, it was assumed that the inter-individual variability of *n*[Pt]/K_d_ was caused only by the [Pt]. The same [Pt] was used for different compounds if they were bound to the same protein. In the present study, the major protein bound to compounds in plasma was α1-acid glycoprotein when a compound’s basic pKa was over 7 (irinotecan, NPC, and APC), and albumin for the others (SN-38 and SN-38G). In blood cells, all compounds except for SN-38G are bound to red blood cells. It was assumed that SN-38G was not transferred to blood cells considering Rb reached the theoretical lower limit value (1 – Hct). Eq. 10 is used to calculate f_b_ of SN-38G.

10 $$ {f}_b=\frac{f_p}{Rb}=\frac{f_p}{1- Hct} $$

(3) **K**_**p**_ **and tissue unbound fraction of compounds**

K_p_ for muscle, skin, adipose, and gut, and tissue unbound fraction (f_h_ and f_gut_) were obtained using in silico calculations (26,27). pKa, clogP, f_p_, and f_b_ were used in these calculations.

(4) **Renal clearance**

Assuming that reabsorption of compounds was negligible, renal clearance (CL_r_) was calculated using Eq. 11:

11 $$ {CL}_r={f}_b\cdot GFR+{CL}_{\sec } $$

where GFR represents glomerular filtration rate and CL_sec_ secretion clearance. CL_sec_ is described in Eqs. 12 to 16 based on the dispersion model.

12 $$ {CL}_{\sec }={Q}_{rtb}\left(1-\frac{4 a}{{\left( a+1\right)}^2\cdot {e}^{\left(\frac{a-1}{2{D}_N}\right)}-{\left( a-1\right)}^2\cdot {e}^{\left(\frac{- a+1}{2{D}_N}\right)}}\right) $$

13 $$ a={\left(1+\frac{4{D}_N\cdot {f}_b\cdot {CL}_{\mathrm{int}, \sec }}{Q_{rtb}}\right)}^{\frac{1}{2}} $$

14 $$ {Q}_{r tb}={Q}_r- GFR $$

15 $$ {Q}_r=\frac{ERPF}{1- Hct} $$

16 $$ ERPF=\frac{GFR}{FF} $$

where CL_int,sec_ represents intrinsic secretion clearance, Q_rtb_ renal uriniferous tubule blood flow, Q_r_ renal blood flow, ERPF effective renal plasma flow, and FF filtration fraction. D_N_ is the dispersion number and is set to 0.17. The average and CV of FF, Hct, and GFR are described in Supplementary Table 3. The CV of CL_int,sec_ that could reproduce the inter-individual variability of CL_r_ for irinotecan, SN-38, SN-38G, and APC observed in a clinical study (36) was estimated, and their average (CV 34.2%), excluding SN-38G, was used for VCS. For SN-38G, the contribution of CL_int,sec_ to CL_r_ is small and its CV values cannot be calculated.

### Performing VCSs and the Comparison of their Outcomes with Clinical Studies

Using a VCS approach, 30 sets of parameters optimized by CNM so that the blood concentration–time data of irinotecan and its metabolites were well described (33), were evaluated whether some sets of parameters can describe well other clinical findings [20 and References 1–17 in Supplementary Text]. The clinical findings showing the similarities to the prediction by VCSs adopted in the present analyses consist of the following 4 categories: (I) the association between SN-38 plasma concentration at the end of infusion (90 min) and UGT1A1 *28 polymorphism or SLCO1B1 c.521T>C polymorphism; (II) the association between neutropenia and each genetic polymorphism of enzymes and transporters; (III) the association between diarrhea and each genetic polymorphism of enzymes and transporters; and (IV) the association between diarrhea and “biliary index (= AUC_(irinotecan)_ × AUC_(SN-38)_ / AUC_(SN-38G)_),” which has been reported as a good biomarker of diarrhea (37). For the categories I, VCS outcomes were compared with those reported by Teft *et al*. (20), which is called the “target study” in this article. The target study was performed with 127 advanced or metastatic cancer patients and focused on 14 genetic polymorphisms of enzymes and transporters, including 6 genetic polymorphisms considered in VCS. For the other categories (Categories II, III, and IV), VCS outcomes were compared not only with the target study, but also with other previously reported studies. The VCS procedure can be described as follows:

1. A virtual population (*n* = 127, equivalent to the number of target study patients) was generated using processes 1–1 and 1–2.
2. 6 genetic polymorphisms (UGT1A1 *28, SLCO1B1 c.521T>C and c.388A>G, ABCG2 c.421C>A, ABCB1 c.3435C>T, and ABCC2 c.-24C>T) among 127 virtual patients were selected.
3. A Monte Carlo simulation was used to generate a value for each parameter following Supplementary Tables 2 and 3.
4. A blood and tissue concentration–time profile was simulated, and the probability of side effects was determined and compared with the findings of the target study. Neutropenia and diarrhea are considered to be related to the AUCs in the plasma and intestinal epithelia, respectively.
5. Processes 1 and 2 were repeated 100 times for different virtual populations to consider the differences among different clinical study results caused by the inter-individual variability of patients.
6. Processes 1 to 3 were repeated for all 30 sets of parameters.

A Welch’s *t* test with post hoc Bonferroni correction was used to compare SN-38 concentration among the polymorphisms. Neutropenia occurred in 21 and diarrhea in 8 patients in the target study. To determine the corresponding values of AUCs in those who suffered from these side effects, we assumed that among the 127 virtual patients, those who had the highest 21 unbound plasma AUCs for SN-38 developed neutropenia, and those who had the highest 8 unbound enterocyte AUCs for SN-38 developed diarrhea. Although side effect factors in the target study were estimated based on logistic regression, none of the factors used by logistic regression were shown in the target study article. Subsequently, Fisher’s extract test was applied to determine the frequency of polymorphism between those with and without side effects. Dominant and recessive genetic models were applied in Fisher’s extract test. The dominant model compares a combination of heterozygous and homozygous variants to the wild type. The recessive model compares a homozygous variant to a combination of heterozygous variants and the wild type. The Wilcoxon rank sum test was used was used to determine the association between the biliary index and diarrhea.

### Analysis of the Effect of the Number of Virtual Patients on the Inter-Individual Variability of Clinical Outcomes

The inter-individual variability of patients may affect the results of clinical studies with insufficient numbers of patients. Thus, the effect of inter-individual variability among virtual patients for the VCS outcomes was compared by performing VCS 100 times with different numbers of virtual patients (25, 39, 51, 64, 96, 127, 192, 256, 384, 512, 1024, and 1280) (Fig. 6). For each VCS, the frequency of a significant (*p* < 0.05) effect of polymorphism of UGT1A1 *28 or SLCO1B1 c.521T>C on the SN-38 plasma concentration at the end of infusion (90 min) was calculated. This analysis was only performed using the set of parameters IDs 1 and 5 as representatives.

### Computational Environment

The simultaneous ordinary differential equations were solved using the MATLAB (MathWorks, Natick, MA) stiff ODE solver ODE15s on MATLAB version 8.6.0 (R2015b). Virtual patients were generated and all statistical tests were performed using R version 3.2.3. All calculations were performed on a workstation (CPU: Xeon E5–2640 v3 × 2; OS: CentOS 6.7 64 bit; RAM: 32 GB).

## Results

### Estimation of the Contribution of CES1 to the Metabolism of Irinotecan to SN-38

**Fig. 3:**
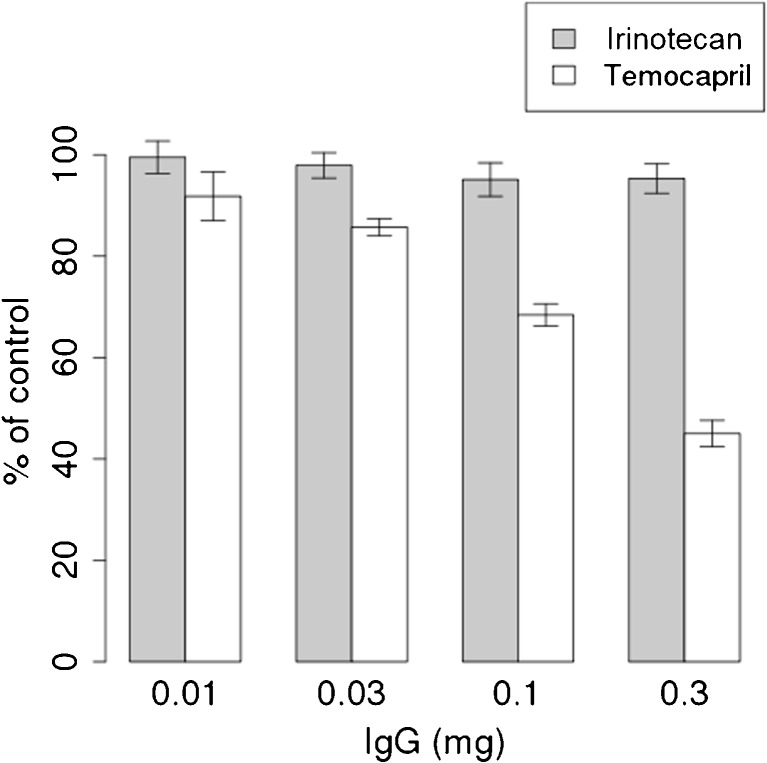
The contribution of CES1 to metabolism of irinotecan and temocapril. The amount of metabolite normalized by control condition is shown with increased amount of CES1A-specific IgG (*N* = 3).

Figure 3 shows the ratio of the production of metabolites (SN-38 from irinotecan and temocaprilat from temocapril) between CES1A IgG-treated and control microsomes (% of control). Temocapril is a well-known substrate of CES1 (38). The ratio for temocapril decreased as the amount of CES1A IgG increased. By using 0.30 mg CES1A IgG, the values were 45.1 ± 2.6 (%) for temocapril and 95.3 ± 2.9 (%) for irinotecan compared with controls, indicating that CES2 plays an important role in the production of SN-38 in human liver.

### Analyses of Pharmacokinetics of Irinotecan and Its Metabolites Based on PBPK Modeling and Optimization of Various Biochemical Parameters Using CNM

**Fig. 4:**
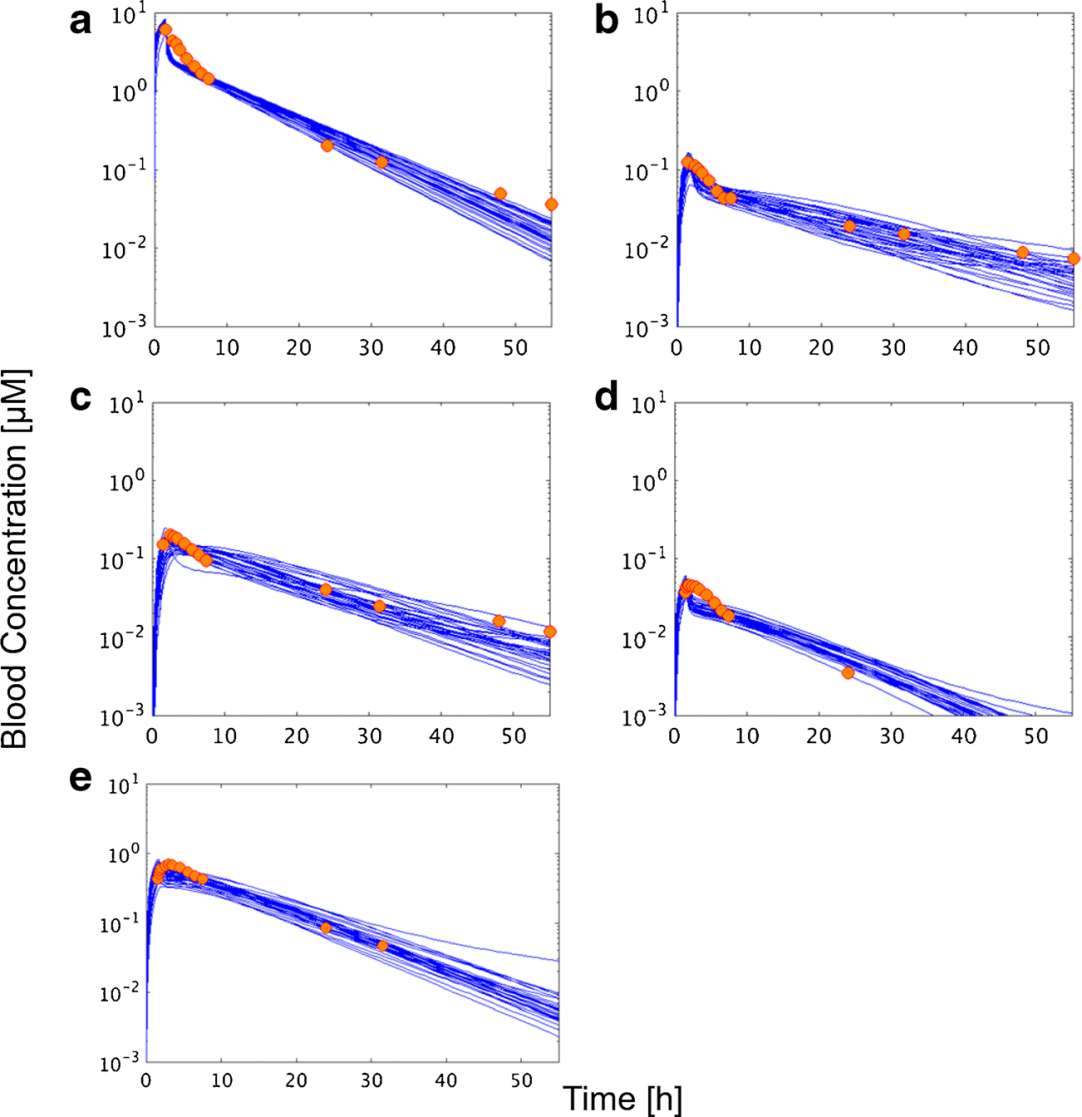
Comparison of clinically obtained blood concentration–time profile of irinotecan (**a**), SN-38 (**b**), SN-38G (**c**), NPC (**d**), and APC (**e**) and the simulated lines obtained using the top 30 sets of parameters optimized by CNM. *Orange dots* represent the observed blood concentration (31). Each *blue line* represents the blood concentration–time profile using a certain set of optimized parameters obtained using CNM. Each figure shows 30 *blue lines* that was obtained by minimizing the weighted sum of squares between predicted and observed concentrations at all sampling points.

Figure 4 shows the blood concentration–time profiles for irinotecan, SN-38, SN-38G, NPC, and APC with 30 sets of parameters optimized by CNM, which minimizes the WSS between simulated and observed concentrations at the various sampling points. The average WSS for each set of parameters is shown in Supplementary Fig. 2, and optimized values for each parameter are shown in Supplementary Table 4. The average WSS for the top 30 sets of parameters ranged from 0.082–0.107.

### Simulation of the Effect of Inter-Individual Variability by Using VCSs

**Fig. 5:**
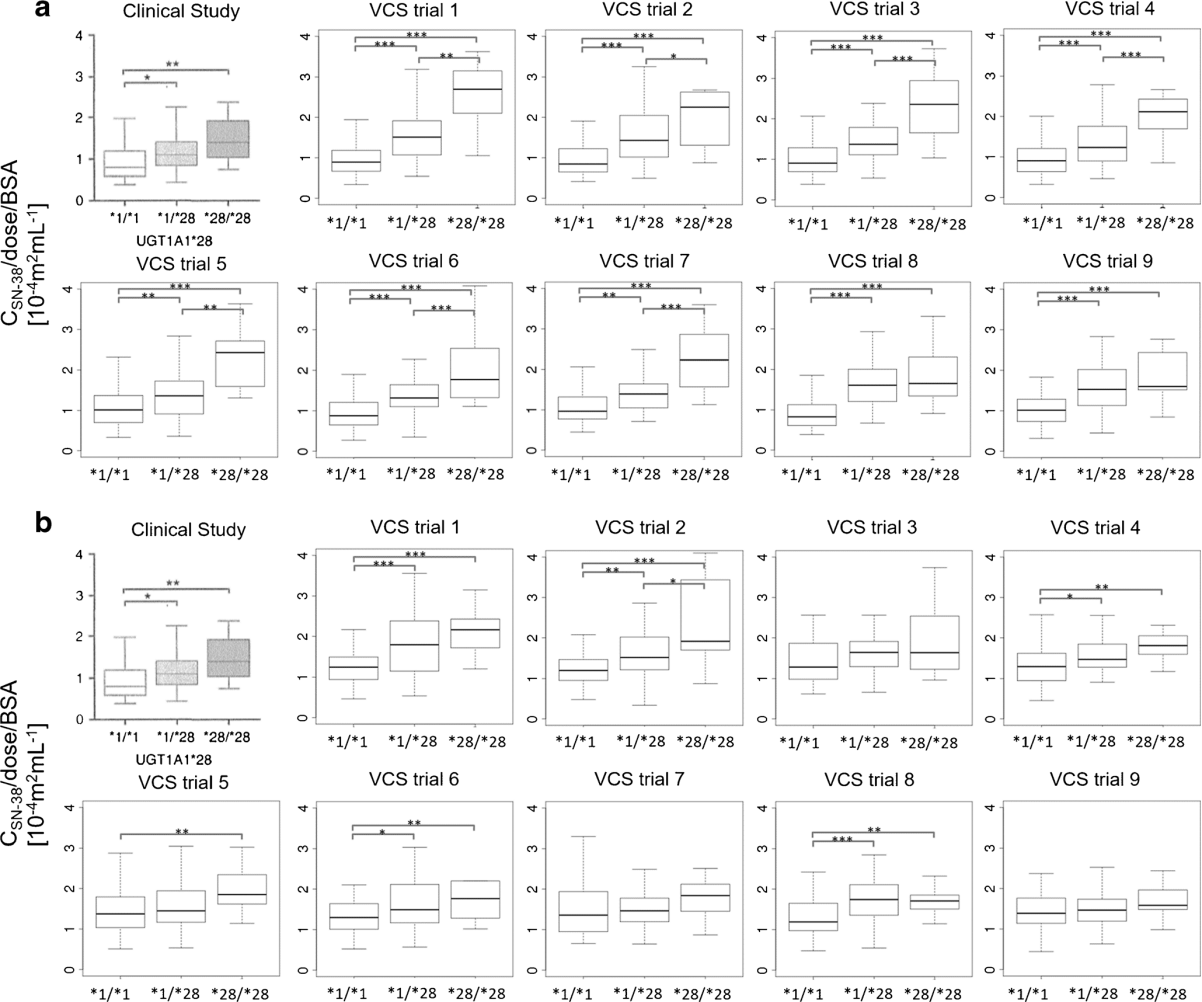

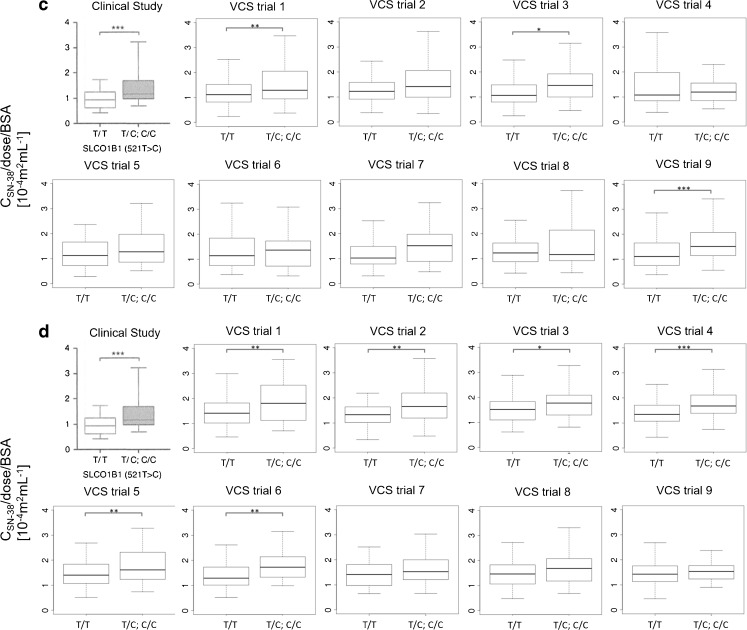
The association between the UGT1A1 *28 or SLCO1B1 c.521T>C polymorphism and dose-normalized SN-38 plasma concentration at the end of infusion (90 min) with 9 VCSs of 100 times of VCSs. (**a**) and (**a**) show the association between the UGT1A1 *28 polymorphism and dose-normalized SN-38 plasma concentration. (**c**) and (**d**) show the association between the SLCO1B1 c.521T>C polymorphism and dose-normalized SN-38 plasma concentration. In (**a**) and (**c**), each virtual patient was generated from the set of parameters ID 1. In (**b**) and (**d**), each virtual patient was generated from the set of parameters ID 5. The top-left panel in each figure shows the results of the target study (20). The others show 9 of the 100 times of VCSs. * *p* < 0.05, ** *p* < 0.01, *** *p* < 0.01.

The VCS was performed 100 times with a different virtual population for each set of parameters. Figure 5 shows the effect of inter-individual variability among the virtual population which comes from other variability than genetic polymorphism. Figure 5a and b show the effect of UGT1A1 *28 polymorphism on the plasma concentration of SN-38 at the end of infusion (90 min) with different populations (ID 1 and ID 5) and Fig. 5 c and d show the effect of SLCO1B1 c.521T>C polymorphism on the plasma concentration of SN-38 at the end of infusion (90 min) with different populations (ID 1 and ID 5). In Fig. 5, the top-left panel in each figure shows the results of the target study (20). The others show 9 of the 100 times of VCSs using the set of parameter ID1 (Fig. 5a and c) and ID 5 (Fig. 5b and D). As shown in Fig. 5a, all cases of VCS could reproduce the result of target study using the set of ID 1. However, using the set of ID 5, Fig. 5b shows that only 5 cases of VCS could reproduce the clinical results. This finding suggests that the same clinical outcomes may not be obtained even if the clinical studies are repeated when the number of patients in the population (n) is small, as in the present case (*n* = 127) with the set of parameters ID 5. In case of Fig. 5c (ID 1) and 5D (ID 5), 3 cases and 6 cases of VCS could reproduce the results of target study for ID 1 and ID 5, respectively. These results suggested that with different set of parameters, different clinical outcomes were predicted.

### The Relationship between the Number of Virtual Patients and the Reproduction Frequency of the Effect of Polymorphisms of UGT1A1 and SLCO 1B1 on the Plasma Concentration of SN 38.

**Fig. 6:**
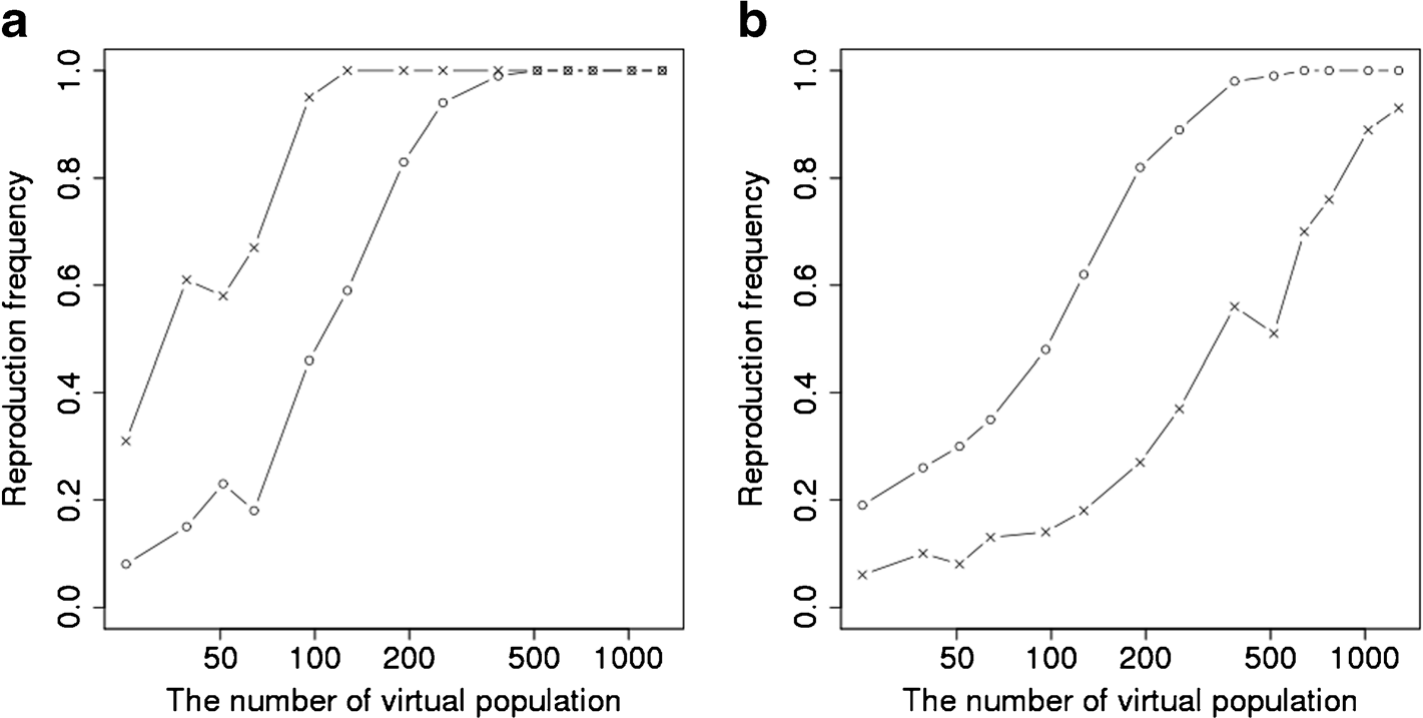
The association between the number of virtual patient and the reproduction frequency of the effect of UGT1A1 *28 or SLCO1B1 c.521T>C polymorphism on dose-normalized SN-38 plasma concentration at the end of infusion (90 min) among 100 times of VCSs. The reproduction frequency of (**a**) shows the effect of UGT1A1 *28 polymorphism on dose-normalized SN-38 plasma concentration. The reproduction frequency of (**b**) shows the effect of SLCO1B1 c.521T>C polymorphism on dose-normalized SN-38 plasma concentration. The set of parameters ID 1 (×) and 5 (○) were used. The number of virtual population in X-axis is shown as a logarithmic scale.

Figure 6 shows the reproduction frequency of the effect of UGT1A1 *28 (Fig. 6a) and SLCO1B1 c.521T>C (Fig. 6b) on the plasma SN-38 concentration at the end of infusion (90 min) among 100 times of VCSs for each number of virtual patient using the set of parameters IDs 1 and 5. The reproduction frequency increased when the number of virtual patients (n) increased, and was higher than 0.8 for *n* = 96 with ID 1 and *n* = 192 with ID 5 for UGT1A1 *28 (Fig. 6a) and *n* = 1024 with ID 1 and *n* = 192 with ID 5 for SLCO1B1 c.521T>C (Fig. 6b).

### Comparison of VCS Outcomes among Sets of Parameters Obtained by CNM to the Target Study with Regard to the Effect of UGT1A1 *28 and SLCO1B1 c.521T>C Genotype on Plasma SN-38 Concentration

**Fig. 7:**
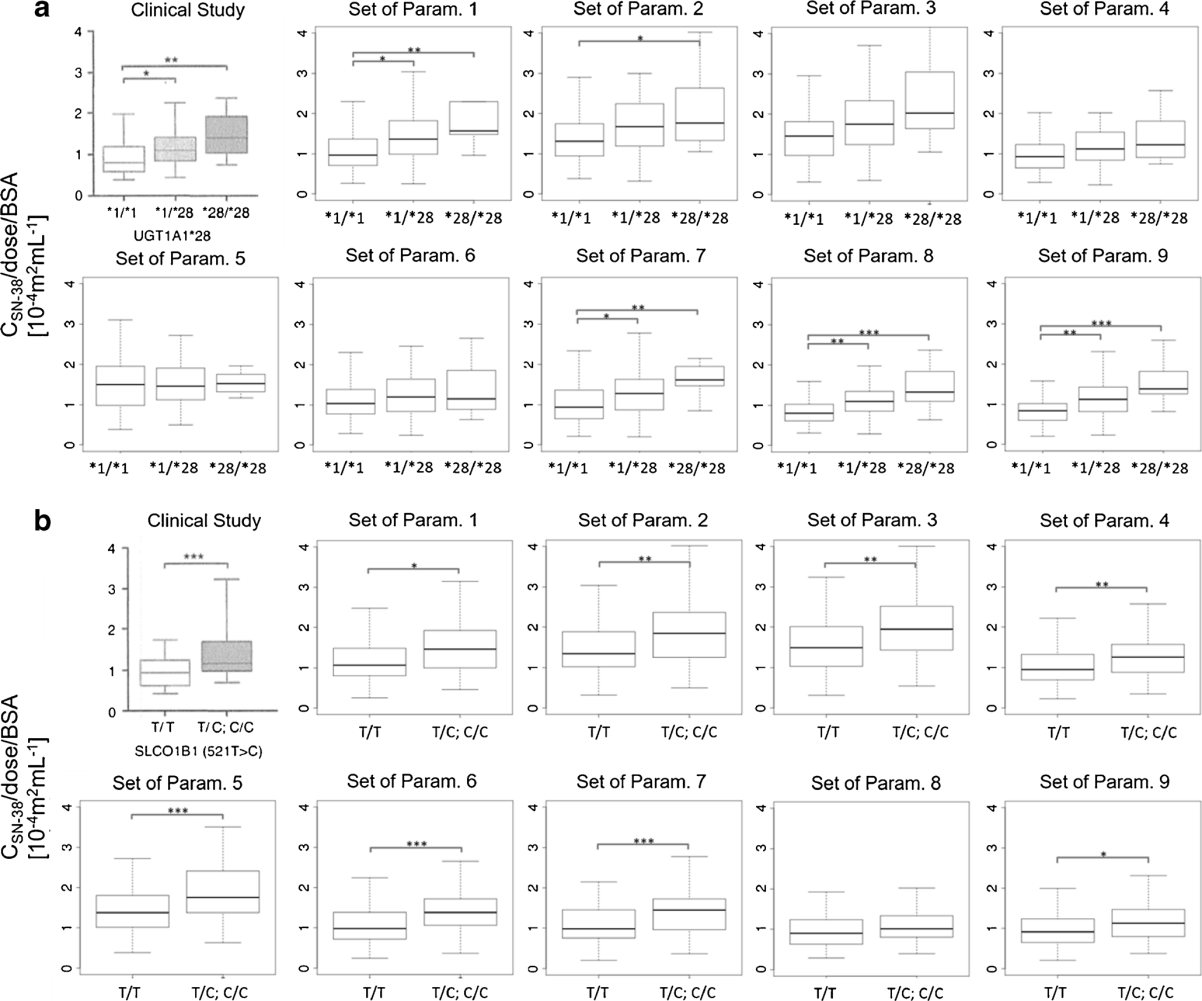
Comparison between clinical study and virtual clinical study outcomes using 9 different sets of parameters determined by CNM. (**a**) and (**b**) show the association between dose-normalized SN-38 plasma concentrations at the end of infusion (90 min) and the UGT1A1*28 genotype (**a**) and SLCO1B1 c.521T>C genotype (**b**). The top-left panel in each figure shows the results of the target study (20). The others show 9 sets of parameters of the 30 sets optimized by CNM. * *p* < 0.05, ** *p* < 0.01, *** *p* < 0.01.

Figure 7 shows that SN-38 plasma concentration at the end of infusion in UGT1A1 *28 (Fig. 7a) and SLCO1B1 c.521T>C (Fig. 7b) genotypes for the target study and 9 VCSs with different sets of parameter values optimized by CNM. The top left shows the clinical results of the target study. The others show 9 VCS outcomes in which a different sets of parameter IDs from 1 to 9 were used (Supplementary Table 4). Figure 7a and b show the results of the same sets of parameters for VCSs. In these VCSs, only the outcomes for the set of parameters ID 1, 7, and 9 could reproduce the results of the target study with regard to the effects of polymorphism of both UGT1A1 and SLCO1B1 on the SN-38 plasma concentrations.

### Selection of the Best Set of Parameters that Could Reproduce the Target Study and Some Other Clinical Studies with Regard to the Effect of UGT1A1 *28 and SLCO1B1 c.521T>C

**Fig. 8:**
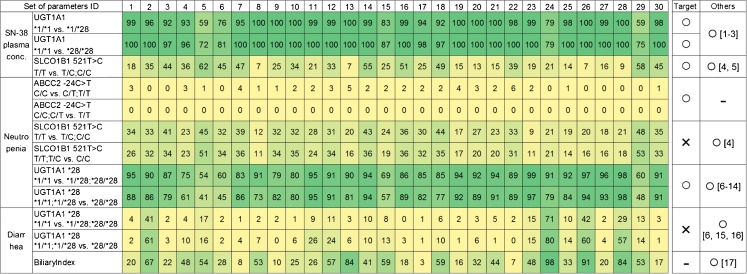
Heat map for the frequency of significance match of the 100 virtual clinical studies to a target clinical study for each of the 30 sets of parameters determined by CNM. Each cell number shows how many times the statistical significance (*p* < 0.05) for each association described in the left row among 100 VCSs. Small frequency of significance described in the cell is colored in yellow, and the higher frequency of significance is colored in green. The “Target” column shows the results of the target study (20). The “Others” column shows the results from other reports than the target study. The references described in “Others” are provided in the Supplementary Text. All the associations except for the association between ABCC2 c.-24C>T genotype and the neutropenia shows some higher frequency of significance between the genetic polymorphisms and SN-38 plasma concentration, neutropenia, or diarrhea. The association between ABCC2 c.-24C>T genotype and the neutropenia is very small if any. This figure shows the results of statistical test. The complete sets of results are described in Supplementary Table 5.

3000 VCSs (100 VCSs multiplied by 30 sets of parameters) were performed. Figure 7 shows only 9 sets of parameters (selected at random) among 30 sets of parameters. Supplementary Table 5 shows all the results of VCSs. Outcomes of frequency of statistical significance of genotypes or phenotype are shown in this table. Some significant associations between the genetic polymorphism of enzymes and transporters and SN-38 plasma concentration and side effects were picked up from Supplementary Table 5 and was shown in Fig. 8. The association in Fig. 8 satisfied either of the 3 conditions described below: (I) the association was statistically significant as shown in the target study; (II) the association between the polymorphism of UGT1A1 *28 or SLCO1B1 c.521T>C and side effects (neutropenia, diarrhea) was statistically significant in other reported studies, though not in the target study; and (III) the side effect diarrhea shows an association with biliary index (37). Some VCSs shown in green color indicated the high similarities between the VCSs and real clinical outcomes. To examine whether the 100 times of VCSs using each set of parameters reproduce the outcomes of clinical studies, the following 7 criteria were used: (I) the effect of UGT1A1 *28 heterozygous on plasma concentration of SN-38 was reproduced over 75 times; (II) the effect of UGT1A1 *28 homozygous on plasma concentration of SN-38 was reproduced over 75 times; (III) the effect of UGT1A1 *28 polymorphism on neutropenia using either dominant model (UGT1A1 *1/*1 *vs.* *1/*28 and *28/*28) or recessive model (UGT1A1 *1/*1 and *1/*28 *vs.* *28/*28) was reproduced over 75 times; (IV) the effect of UGT1A1 *28 polymorphism on diarrhea using either dominant model or recessive model was reproduced over 25 times; (V) the effect of SLCO1B1 c.521T>C on plasma concentration of SN-38 was reproduced over 25 times; (VI) the effect of SLCO1B1 c.521T>C on neutropenia using either dominant model (SLCO1B1 521 T/T *vs.* T/C and C/C) or recessive model (SLCO1B1 521 T/T and T/C *vs.* C/C) was reproduced over 25 times; and (VII) the association between biliary index and diarrhea was reproduced over 25 times. Under this definition, 19 sets of parameters were satisfactory in terms of the consistency of more than or equal to 5 of 7 criteria. Especially, ID 2, 10, 11, 12, 14, and 18 were satisfactory with consistency of more than 5 criteria.

## Discussion

The purpose of this study was to establish a PBPK model that could be used to obtain insight into the factors associated with the side effects caused by irinotecan. First, the key findings were briefly described. The parameters for the PBPK model were estimated as the first step in our analyses. Because it was difficult to estimate a large number of unknown parameters, CNM was newly employed to optimize unknown parameters. By using CNM, over 30 sets of parameters, which could reproduce not only the average blood concentration–time profile of irinotecan and its metabolites, but also urinary and fecal accumulation–time profiles (24), were successfully obtained. The sets of parameters obtained were also evaluated in terms of whether they could reproduce the results of reported clinical outcomes of irinotecan-induced side effects such as neutropenia and delayed diarrhea using a VCS approach. The VCS results indicated three important findings: (I) inter-individual variability of patients has the potential to cause a different clinical outcome with a small number of patients; *n* ≥ 192 is needed to obtain a significant result with the effect of SLCO1B1 c.521T>C on the plasma concentration of SN-38, (II) specific parameters were strongly correlated with the frequency of reproducing clinical observations, and (III) the biliary index is considered to be a better biomarker than UGT1A1 *28 polymorphism regarding susceptibility to diarrhea.

### PBPK Modeling of Irinotecan and Its Metabolites

First, the contribution of CES1 to the metabolism of irinotecan to SN-38 in the liver was estimated using CES1A IgG. As shown in Fig. 3, it was found that CES2 plays a key role in the metabolism of irinotecan to SN-38 in the liver. The relative expression level of mRNA for CES2 to CES1 in the intestine is higher than that in the liver (39), suggesting that the contribution of CES1 to the metabolism of irinotecan is considered small if any in the liver and intestine. Therefore, the CES1 genotype was not considered in the VCS.

Figure 4 shows that multiple sets of parameters in the PBPK model of irinotecan and its metabolites that could reproduce the observed blood concentration–time profiles. This PBPK-based optimization was obtained using a CNM (18). Although the same optimization using a Levenberg–Marquardt method, which is a conventional fitting approach, was performed on MATLAB, parameters were not converged because of too many unknown parameters. The computational time for CNM optimization with the initial 10,000 sets of parameters was about 15 min. This result shows that CNM is a powerful algorithm by which to find multiple sets of parameters for PBPK models.

Yoshida *et al*. (19) reported estimated PBPK model parameters for the metabolism of irinotecan and its metabolites that reproduced observed urinary and fecal accumulation–time profiles (24) using CNM. Urinary and fecal accumulation was also simulated using the 30 sets of parameters obtained using CNM (Supplementary Fig. 3). Considering the variance of the observed accumulation, the simulated accumulation profiles using these set s of parameters are considered to be comparable with the observed accumulation–time profiles.

### VCSs Using Sets of Parameters Obtained by CNM to Examine whether the Clinical Outcome with Regard to the Effect of Genetic Polymorphism on the SN-38 Plasma Concentrations and Side Effects Can be Produced

As shown in Fig. 5, even when the virtual populations were generated from the same average value for all parameters, different results of VCS with different virtual populations were obtained. This result indicates that inter-individual variability of patients has the potential to cause a different clinical outcome with such a small number of patients as the 127 adopted in the target clinical study (20). To calculate the effect of inter-individual variability, 100 VCSs were performed with different numbers of virtual patients. Figure 6 shows the reproduction frequency of the plasma SN-38 concentration at the end of infusion (90 min) among 100 VCSs with regard to the effect of UGT1A1 *28 (Fig. 6 a) and SLCO1B1 c.521T>C (Fig. 6 b), which was estimated by VCSs changing the number of virtual patients in each clinical study. In Fig. 6, the reproduction frequency increases according to the increase in the number of virtual population. In this analysis, the reproduction frequency is an index similar to statistical power. The statistical power of 0.8 has been often used as the threshold of its power. When 0.8 was applied as the threshold of reproduction frequency, the reproduction frequency was higher than 0.8 at *n* = 192 for SLCO 1B1 and ID 5, at *n* = 96 for UGT1A1 and ID 1 (Fig. 6). ID 5 used in this analysis could reproduce the effect of SLCO1B1 polymorphism on the plasma SN-38 concentration with the highest frequency in all sets of parameters for *n* = 127 (Fig. 8). On the other hand, many sets of parameters could reproduce the effect of UGT1A1 polymorphism with the highest frequency (Fig. 8). It was thus estimated that *n* ≥ 192 is needed to obtain the significant result of the effect of SLCO1B1 c.521T>C. Generally, it is difficult to perform a clinical study using such large number of patients. On the other hand, large-scale VCSs can overcome the effect of inter-individual variability and provide a good estimate of true clinical outcomes.

Some clinical studies have reported that the UGT1A1*28 genotype does not affect the frequency of neutropenia (40), even if many other studies have reported this effect. Figure 8 shows that the impact of UGT1A1*28 polymorphism on increasing the plasma SN-38 concentration and side effects; neutropenia was reproduced in more than 80 of the 100 times of VCS trials with most sets of parameters except for ID 4, 5, 6, 15, 24, and 29. The common features of parameters among ID 4, 5, 6, 15, 24, and 29 were searched, and these 6 sets were found to have the lowest values for the ratio (f_glu(SN-38)_) of metabolic clearance for glucuronidation of SN-38 to hepatic intrinsic clearance. Then, the correlation of f_glu(SN-38)_ and the reproduction frequency of the impact of UGT1A1*28 polymorphism on increasing the neutropenia was plotted using 30 sets of parameters (Supplementary Fig. 4A). Supplementary Fig. 4A shows that each set of parameters in which f_glu(SN-38)_ was more than 0.8 had a high reproduction frequency of more than 0.8. This is because the effect of UGT1A1 *28 is less pronounced when the f_glu(SN-38)_ value becomes smaller. Similarly, when the correlation of R_dif,h(SN-38)_ and the reproduction frequency of the impact of SLCO1B1 c.521T>C polymorphism on increasing the neutropenia was plotted using 30 sets of parameters (Supplementary Fig. 4B), the set of parameters having a large R_dif,h(SN-38)_ could not reproduce the effect of SLCO1B1 c.521T>C on neutropenia with high frequency. Because the contribution of SLCO1B1 to the total hepatic uptake clearance becomes smaller with larger R_dif,h(SN-38)_, SLCO1B1 c.521T>C polymorphism had little effect on SN-38 plasma exposure.

The target study also found that only heterozygous ABCC2 c.-24C>T, but not homozygous carriers reduced the risk of neutropenia (Fig. 8). As seen in Fig. 8, no set of parameters could reproduce this result in more than 10 of the 100 times of VCSs. We believe that the target study may have obtained this result by chance, considering only heterozygous carriers achieved statistical significance. In association with diarrhea, the target study found that a common SNP in CES1 (rs2244613) caused a lower risk. However, the current study (Fig. 1) suggests that CES2 mainly contributes to the metabolism of irinotecan to SN-38 in the liver and intestine, as discussed previously. The mechanism by which the target study found the effect of CES1 rs2244613 on the occurrence of diarrhea has not yet been clarified.

Gupta *et al*. (37) proposed that patients with a high biliary index have a risk of diarrhea. By contrast, other clinical studies have reported that UGT1A1 *28 polymorphism increases the risk of diarrhea (4,5). Figure 8 includes the results of tests according to the association between these factors and diarrhea for 100 times of VCSs among 30 sets of parameters. The VCSs were performed by assuming the diarrhea is influenced by the unbound AUC of SN-38 in the intestinal epithelia. As shown in Fig. 8, the sets of parameters for which the frequency of statistical significance of association of UGT1A1 *28 polymorphism with diarrhea is high also exhibited a high frequency of significant association between the biliary index and diarrhea. This result may be explained by the possibility that UGT1A1 *28 polymorphism affects the increase in the biliary index, which has been found clinically (41). However, the frequency of significant association between the biliary index and diarrhea was higher than that of the association between UGT1A1 *28 polymorphism and diarrhea for all sets of parameters (Fig. 8). Consequently, the biliary index is considered to be a better biomarker than UGT1A1 *28 for susceptibility of diarrhea.

### Perspective of VCS

In the present study, VCS was performed to evaluate whether reported clinical studies could be reproduced by the PBPK model, which was a “retrospective” approach. In the practical application of VCS to new drug development, we have to use a “prospective” approach. What kind of procedure should be adopted for this purpose? A PBPK model is first established for a candidate compound after a Phase I clinical study, using observed plasma concentration–time profile data together with many available *in vitro* experimental data on the binding, metabolism and transport. We then can estimate the exposure of the compound in the target tissues as a function of dose in terms of pharmacological and side effects. Next, the relationship between the simulated target tissue exposure of the compound and the pharmacological effects and side effects are simulated. To do so, we have to estimate the exposure to cause pharmacological effects and side effects using *in vivo* and *in vitro* data. Those may include clinical phase I studies (in the case of anticancer drugs) and *in vitro* experiments using human normal cells, cancer cells, gene expressed and knockdown cells and *in vivo* animal model experiments. These studies will provide the thresholds of exposure, i.e., the criteria as to whether effects or side effects occur. Then, various virtual populations (e.g., genetic polymorphism, ethnicity and disease state) are generated. Finally, after VCS is performed using a generated varied virtual population, the PBPK model is developed and the relationships of target tissue exposure to the effects and side effects of the compound are estimated. Finally, we will be able to estimate the occurrence probability of effects and side effects and the suitable dosage for every virtual population such as population with different genetic polymorphisms, co-administered drugs, different ethnicity and dysfunctions of organs (liver, kidney).

We generated 1,000,000 virtual people using the ID 2 set of parameters as average values and variabilities (shown as CV) as shown in Supplementary Tables 3 and 4, and simulated unbound AUC values of SN-38 in the plasma and enterocyte. The thresholds of neutropenia and diarrhea were set for plasma unbound AUC > 26.35 [nM⋅h] and enterocyte unbound AUC > 53.60 [nM⋅h], respectively, the values of which were determined as average thresholds for 100 times of VCSs using the ID 2 set of parameters. Thus, the probability of neutropenia and diarrhea were estimated by VCS with 1,000,000 virtual people (Supplementary Fig. 5). As a result, it was estimated that 16.3% and 6.04% of patients would experience neutropenia and diarrhea, respectively. The occurrence frequency of neutropenia and diarrhea were reported to be 23.0 ± 3.4% and 10.3 ± 3.0%, respectively, by summarizing previous clinical studies (Supplementary Table 6). Comparing the frequency of side effects obtained from VCS to that of reported studies, VCS provided somewhat lower values, probably because thresholds values were determined based only on the results of the target study.

## Conclusion

This work successfully obtained multiple sets of PBPK model parameters that could reproduce the effects of genetic polymorphisms of UGT1A1 *28 and SLCO1B1 c.521T>C on the plasma concentration of SN-38 and side effects such as neutropenia and diarrhea using a VCS approach. To optimize the numerous biochemical parameters of irinotecan and its metabolites in the PBPK model, a CNM parameter optimization algorithm was introduced. VCS suggested that inter-individual variabilities affect clinical outcomes when performed with smaller number of patients. The current VCS confirmed the importance of the biliary index as a better biomarker of irinotecan-induced diarrhea compared with only UGT1A1 *28 polymorphism. By contrast, VCS suggested that the importance of polymorphism of other factors (CES1, ABCC2, ABCB1, and ABCG2) should be interpreted carefully.

## Electronic supplementary material

ESM 1 (DOCX 23.4 kb)

Supplementary Figure 1 **Uptake of SN-38G by OATP1B1 (** ***N*** **= 4).** (●) and (○) and represent the uptake of SN-38G by HEK293/OATP1B1 and HEK293/mock cells, respectively. 0.3 μM SN-38G was used. * represents the *p* < 0.05 (Welch’s t-test) comparing the uptake amount of SN-38G between HEK293/OATP1B1 and HEK293/mock cells for each time point. The uptake of SN-38G at 15 min is 6.35 ± 0.54 (HEK293/OATP1B1) and 2.35 ± 0.71 (HEK293/mock) μL/mg protein. (JPEG 35 kb)

High resolution image (TIFF 1321 kb)

Supplementary Figure 2 **Association between the average WSS and the sets of parameters obtained using CNM**. The top 100 sets of parameters of 1,000,000 sets sorted in ascending order of WSS are shown. (JPEG 33 kb)

High resolution image (TIFF 58 kb)

Supplementary Figure 3 **Validation of the reproducibility for reported mass balance obtained in clinical study** (24) **using the top 30 sets of parameters.** Orange dots represent the observed values that are calculated from the relative abundance of radioactive compounds in urine and feces. Each blue line represents the total amount time profile of urine (A) and feces (B). (JPEG 66 kb)

High resolution image (TIFF 293 kb)

Supplementary Figure 4 **Correlation among 30 sets of parameters between f**_**glu(SN-38)**_ **(A) or R**_**dif,h(SN-38)**_ **(B) and the frequency of reproduction of the association between neutropenia and genetic polymorphism.** (A) shows the relationship between f_glu(SN-38)_ for 30 sets of parameters and the frequency of the association between the effect of UGT1A1 *28 polymorphism and neutropenia using lager frequency cases; either dominant model (UGT1A1 *1/*1 *vs.* *1/*28 and *28/*28) or recessive model (UGT1A1 *1/*1 and *1/*28 *vs.* *28/*28) (B) shows the relationship between R_dif,h(SN-38)_ for 30 sets of parameters and the frequency of a significant association between the effect of SLCO1B1 c.521T>C polymorphism and neutropenia using lager frequency cases; either dominant model (SLCO1B1 521 T/T *vs.* T/C and C/C) or recessive model (SLCO1B1 521 T/T and T/C *vs.* C/C). The R_dif,h(SN-38)_ in X-axis of (B) is shown as a logarithmic scale. (JPEG 48 kb)

High resolution image (TIFF 257 kb)

Supplementary Figure 5 **Histogram of unbound plasma (A) and enterocyte (B) AUC of SN-38 among 1,000,000 virtual patients.** Dotted line shows the threshold AUC for neutropenia and diarrhea (neutropenia, plasma unbound AUC > 26.35 [nM⋅h]; diarrhea, enterocyte unbound AUC > 53.60 [nM⋅h]). (JPEG 54 kb)

High resolution image (TIFF 424 kb)

Supplementary Table 1 (DOCX 32 kb)

Supplementary Table 2 (DOCX 21 kb)

Supplementary Table 3 (DOCX 27 kb)

Supplementary Table 4 (DOCX 42 kb)

Supplementary Table 5 (XLSX 16 kb)

Supplementary Table 6 (DOCX 20 kb)

## Abbreviations

AUC: Area under the curve of concentration time profile
BCRP: Breast cancer resistance protein
CES: Carboxylesterase
CL: Clearance
CNM: Cluster Newton method
CYP: Cytochrome P450
IgG: Immunoglobulin G
MDR1: Multiple drug resistance 1
MRP2: Multidrug resistance-associated protein 2
OATP: Organic anion transporting polypeptide
PBPK: Physiologically-based pharmacokinetics
PS: Permeability surface area-product
SN-38: 7-Ethyl-10-hydroxy-camptothecin
SN-38G: 7-Ethyl-10-hydroxy-camptothecin glucuronide
UGT: Uridine diphosphate glucuronosyltransferases
VCS: Virtual clinical study
WSS: Weighted sum of squares
