# Supplementary material for: Virtual Clinical Studies to Examine the Probability Distribution of the AUC at Target Tissues Using Physiologically-Based Pharmacokinetic Modeling: Application to Analyses of the Effect of Genetic Polymorphism of Enzymes and Transporters on Irinotecan Induced Side Effects
Source: Pharm Res. 2017 Apr 10;34(8):1584–600. doi: 10.1007/s11095-017-2153-z (PMC5498655; doi:10.1007/s11095-017-2153-z)
Supplement: Supplementary file 1 — (DOCX 23.4 kb) [file 11095_2017_2153_MOESM1_ESM.docx]

**Supplementary Text**

**Nomenclature**

AUC, area under the blood concentration-time curve; C, total (unbound+bound) concentration of irinotecan, SN-38, SN-38G, NPC, or APC; C_tissue(compound),_ total (unbound+bound) tissue concentration of compound; CL_int,all_, hepatic overall intrinsic clearance; CL_bile_, intrinsic clearance of biliary excretion; CL_met,ent_, intrinsic clearance of intestinal metabolism; CL_met,h_, intrinsic clearance of hepatic metabolism; CL_compound1,tissue_,_(compound2)_, intrinsic metabolic clearance from compound2 to compound1; CLr, renal clearance; EHC, enterohepatic circulation; F_a_F_g_, intestinal availability; f_b_, protein unbound fraction in blood; f_h_, hepatic protein unbound fraction; f_gut_, protein unbound fraction in the enterocyte; HC, hepatocytes; HE, hepatic extracellular space; k_a_, absorption rate constant; k_bile_, transit rate constant for EHC; k_dec_, deconjugated rate constant from SN-38G to SN-38; k_feces_, fecal excretion rate constant; K_p_, tissue to blood concentration ratio; PBPK, physiologically-based pharmacokinetic; muc,b, mucosal blood; PS_act,inf,h_, active uptake intrinsic clearance on sinusoidal membrane in the liver; PS_act,eff,ent_, active efflux intrinsic clearance on sinusoidal membrane in the enterocyte; PS_dif,inf,ent_, influx intrinsic clearance by passive diffusion through sinusoidal membrane in the enterocyte; PS_dif,inf,h_, influx intrinsic clearance by passive diffusion through sinusoidal membrane in the liver; PS_dif,eff,ent_, efflux intrinsic clearance by passive diffusion through sinusoidal membrane in the enterocyte; PS_dif,eff,h_, efflux intrinsic clearance by passive diffusion through sinusoidal membrane in the liver; Q_tissue_, blood flow rate in tissue; V_tissue_, tissue volume; WSS, weighted sum of squares; X, total (unbound+bound) amount of irinotecan, SN-38, SN-38G, NPC, or APC

**Ordinary differential equations**

**Central compartment**

V_central_ * dC_central_ / dt = Q_h_ * C_HE5_ + Q_muscle_ * (C_muscle_ / K_p,muscle_ – C_central_) + Q_skin_ * (C_skin_ / K_p,skin_ – C_central_) + Q_adipose_ * (C_adipose_ / K_p,adipose_ – C_central_) – CL_r_ * C_central_ – Q_h_ * C_central_

**Distribution compartments (Muscle, Skin, Adipose, and Serosa)**

V_muscle_ * dC_muscle_ / dt = Q_muscle_ * (C_central_ - C_muscle_ / K_p,muscle_)

V_skin_ * dC_skin_ / dt = Q_skin_ * (C_central_ - C_skin_ / K_p,skin_)

V_adipose_ * dC_adipose_ / dt = Q_adipose_ * (C_central_ - C_adipose_ / K_p,adipose_)

V_serosa_ * dC_serosa_ / dt = Q_serosa_ * (C_central_ – C_serosa_ / K_p,gut_)

**Hepatic extracellular compartments (HE,i; i = 1 to 5)**

(V_HE_ / 5) * dC_HE,i_ / dt = (Q_h_ – Q_serosa_ – Q_mucosa_) * C_central_ + Q_serosa_ * C_serosa_ / K_p,gut_ + Q_mucosa_ * C_muc,b_ – Q_h_ * C_HE,i_ + f_b_ * ((PS_act,inf,h_ + PS_dif,inf,h_) / 5) * C_HE,i_ + f_h_ * (PS_dif,eff,h_ / 5) * C_HC,i_ (i = 1)

(V_HE_ / 5) * dC_HE,i_ / dt = Q_h_ * (C_HE,i-1_ – C_HE,i_) + f_b_ * ((PS_act,inf,h_ + PS_dif,inf,h_) / 5) * C_HE,i_ + f_h_ * (PS_dif,eff,h_ / 5) * C_HC,i_ (i = 2 to 5)

(PS_act,inf,h_ = 0 for irinotecan, NPC, and APC)

**Hepatocyte compartments (HC,i; i = 1 to 5)**

(V_HC_ / 5) * dC_HC,i_ / dt = fb * ((PS_act,inf,h_ + PS_dif,inf,h_) / 5) * C_HE,i_ – f_h_ * ((PS_dif,eff,h_ + CL_met,h +_ CL_bile_) / 5) * C_HC,i_ + X_i_(t)

where

CL_met,h_ = CL_SN-38,h(irinotecan)_ + CL_NPC,h(irinotecan)_ + CL_APC,h(irinotecan)_ + CL_others,h(irinotecan)_ for irinotecan

CL_SN-38G,h(SN-38)_ for SN-38

0 for SN-38G, NPC, and APC,

X_i_(t) = 0 for irinotecan,

CL_SN-38,h(irinotecan)_ * C_HC,i(irinotecan)_ + CL_SN-38,h(NPC)_ * C_HC,i(NPC)_ for SN-38,

CL_SN-38G,h(SN-38)_ * C_HC,i(SN-38)_ for SN-38G,

CL_NPC,h(irinotecan)_ * C_HC,i(irinotecan)_ for NPC,

CL_APC,h(irinotecan)_ * C_HC,i(irinotecan)_ for APC

**EHC compartments (EHC)**

dX_EHC,1_ / dt = f_h_ * (CL_bile_ / 5) * (C_HC,1_ + C_HC,2_ + C_HC,3_ + C_HC,4_ + C_HC,5_) – k_bile_ * X_EHC,1_

dX_EHC,2_ / dt = k_bile_ * (X_EHC,1_ - X_EHC,2_)

dX_EHC,3_ / dt = k_bile_ * (X_EHC,2_ - X_EHC,3_)

**Gut compartments**

dX_intestine_ / dt = k_bile_ * X_EHC,3_ + f_gut_ * (AR * PS_dif,eff,ent_ + PS_act,eff,ent_) * C_enterocyte_ – (k_a_ + k_feces_) * X_intestine_ + Y(t)

where

Y(t) = 0 for irinotecan, NPC, and APC,

k_dec_ * X_intestine(SN-38G)_ for SN-38,

–k_dec_ * X_intestine(SN-38G)_ for SN-38G

V_enterocyte_ * dC_enterocyte_ / dt = k_a_ * X_intestine_ + f_b_ * PS_dif,inf,ent_ * C_muc,b_ – f_gut_ * {(AR + 1) * PS_dif,eff,ent_ + CL_met,ent_ + PS_act,eff,ent_} * C_enterocyte_ + Z(t)

where

CL_met,ent_ = CL_SN-38,ent(irinotecan)_ + CL_NPC,ent(irinotecan)_ + CL_APC,ent(irinotecan)_ + CL_others,ent(irinotecan)_ for irinotecan

CL_SN-38G,ent(SN-38)_ for SN-38

0 for SN-38G, NPC, and APC,

Z(t) = 0 for irinotecan,

CL_SN-38,ent(irinotecan)_ * C_enterocyte(irinotecan)_ + CL_SN-38,ent(NPC)_ * C_enterocyte(NPC)_ for SN-38,

CL_SN-38G,ent(SN-38)_ * C_enterocyte(SN-38)_ for SN-38G,

CL_NPC,ent(irinotecan)_ * C_enterocyte(irinotecan)_ for NPC,

CL_APC,ent(irinotecan)_ * C_enterocyte(irinotecan)_ for APC

V_muc,b_ * dC_muc,b_ / dt = Q_mucosa_ * C_central_ + f_gut_ * PS_dif,eff,ent_ * C_enterocyte_ – Q_mucosa_ * C_muc,b_ – f_b_ * PS_dif,inf,ent_ * C_muc,b_

**Excretion compartments**

dX_feces_ / dt = k_feces_ * X_intestine_

dX_urine_ / dt = CL_r_ * C_central_

**Optional equation**

PS_act,inf_ = CL_int,all_ / β / (1 + R_dif_)

PS_dif,inf_ = PS_act,inf_ * R_dif_

PS_dif,eff_ = PS_dif,inf_

CL_met,h_ = PS_dif,eff_ * β / (1 – β) * (1 – f_bile_)

CL_bile_ = PS_dif,eff_ * β / (1 – β) * f_bile_

R_dif,ent_ = PS_act,eff,ent_ / (AR * PS_dif,eff,ent_)

R_met,CYP3A_ = CL_NPC,h(irinotecan)_ / CL_NPC,ent(irinotecan)_ = CL_APC,h(irinotecan)_ / CL_APC,ent(irinotecan)_

R_met,CES_ = CL_SN-38,h(irinotecan)_ / CL_SN-38,ent(irinotecan)_ = CL_SN-38,h(NPC)_ / CL_SN-38,ent(NPC)_

SAR = PS_dif,eff(irinotecan)_ / PS_dif,eff,ent(irinotecan)_ = PS_dif,eff(SN-38)_ / PS_dif,eff,ent(SN-38)_ = PS_dif,eff(SN-38G)_ / PS_dif,eff,ent(SN-38G)_ = PS_dif,eff(NPC)_ / PS_dif,eff,ent(NPC)_ = PS_dif,eff(APC)_ / PS_dif,eff,ent(APC)_

**Optional inequality**

R_met,CYP3A_ > 1

R_met,CES_ > 1

CL_SN-38G,h(SN-38)_ > CL_SN-38G,ent(SN-38)_

**References in Figure 8**

1. Cai X, Cao W, Ding H, Liu T, Zhou X, Wang M, Zhong M, Zhao Z, Xu Q, Wang L. Analysis of UGT1A1*28 genotype and SN-38 pharmacokinetics for irinotecan-based chemotherapy in patients with advanced colorectal cancer: results from a multicenter, retrospective study in Shanghai. J Cancer Res Clin Oncol. 2013;139(9):1579-89.

2. Ramchandani RP, Wang Y, Booth BP, Ibrahim A, Johnson JR, Rahman A, Mehta M, Innocenti F, Ratain MJ, Gobburu JV. The role of SN-38 exposure, UGT1A1*28 polymorphism, and baseline bilirubin level in predicting severe irinotecan toxicity. J Clin Pharmacol. 2007;47(1):78-86.

3. Iyer L, Das S, Janisch L, Wen M, Ramírez J, Karrison T, Fleming GF, Vokes EE, Schilsky RL, Ratain MJ. UGT1A1*28 polymorphism as a determinant of irinotecan disposition and toxicity. Pharmacogenomics J. 2002;2(1):43-7.

4. Han JY, Lim HS, Park YH, Lee SY, Lee JS. Integrated pharmacogenetic prediction of irinotecan pharmacokinetics and toxicity in patients with advanced non-small cell lung cancer. Lung Cancer. 2009;63(1):115-20.

5. Innocenti F, Kroetz DL, Schuetz E, Dolan ME, Ramírez J, Relling M, Chen P, Das S, Rosner GL, Ratain MJ. Comprehensive pharmacogenetic analysis of irinotecan neutropenia and pharmacokinetics. J Clin Oncol. 2009;27(16):2604-14.

6. Martinez-Balibrea E, Abad A, Martínez-Cardús A, Ginés A, Valladares M, Navarro M, Aranda E, Marcuello E, Benavides M, Massutí B, Carrato A, Layos L, Manzano JL, Moreno V. UGT1A and TYMS genetic variants predict toxicity and response of colorectal cancer patients treated with first-line irinotecan and fluorouracil combination therapy. Br J Cancer. 2010;103(4):581-9.

7. Ruzzo A, Graziano F, Loupakis F, Santini D, Catalano V, Bisonni R, Ficarelli R, Fontana A, Andreoni F, Falcone A, Canestrari E, Tonini G, Mari D, Lippe P, Pizzagalli F, Schiavon G, Alessandroni P, Giustini L, Maltese P, Testa E, Menichetti ET, Magnani M. Pharmacogenetic profiling in patients with advanced colorectal cancer treated with first-line FOLFIRI chemotherapy. Pharmacogenomics J. 2008;8(4):278-88.

8. Cecchin E, Innocenti F, D'Andrea M, Corona G, De Mattia E, Biason P, Buonadonna A, Toffoli G. Predictive role of the UGT1A1, UGT1A7, and UGT1A9 genetic variants and their haplotypes on the outcome of metastatic colorectal cancer patients treated with fluorouracil, leucovorin, and irinotecan. J Clin Oncol. 2009;27(15):2457-65.

9. Rouits E, Charasson V, Pétain A, Boisdron-Celle M, Delord JP, Fonck M, Laurand A, Poirier AL, Morel A, Chatelut E, Robert J, Gamelin E. Pharmacokinetic and pharmacogenetic determinants of the activity and toxicity of irinotecan in metastatic colorectal cancer patients. Br J Cancer. 2008;99(8):1239-45.

10. Innocenti F, Undevia SD, Iyer L, Chen PX, Das S, Kocherginsky M, Karrison T, Janisch L, Ramírez J, Rudin CM, Vokes EE, Ratain MJ. Genetic variants in the UDP-glucuronosyltransferase 1A1 gene predict the risk of severe neutropenia of irinotecan. J Clin Oncol. 2004;22(8):1382-8.

11. Minami H, Sai K, Saeki M, Saito Y, Ozawa S, Suzuki K, Kaniwa N, Sawada J, Hamaguchi T, Yamamoto N, Shirao K, Yamada Y, Ohmatsu H, Kubota K, Yoshida T, Ohtsu A, Saijo N. Irinotecan pharmacokinetics/pharmacodynamics and UGT1A genetic polymorphisms in Japanese: roles of UGT1A1*6 and *28. Pharmacogenet Genomics. 2007;17(7):497-504.

12. Yamamoto N, Takahashi T, Kunikane H, Masuda N, Eguchi K, Shibuya M, Takeda Y, Isobe H, Ogura T, Yokoyama A, Watanabe K. Phase I/II pharmacokinetic and pharmacogenomic study of UGT1A1 polymorphism in elderly patients with advanced non-small cell lung cancer treated with irinotecan. Clin Pharmacol Ther. 2009;85(2):149-54.

13. Glimelius B, Garmo H, Berglund A, Fredriksson LA, Berglund M, Kohnke H, Byström P, Sørbye H, Wadelius M. Prediction of irinotecan and 5-fluorouracil toxicity and response in patients with advanced colorectal cancer. Pharmacogenomics J. 2011;11(1):61-71.

14. Côté JF, Kirzin S, Kramar A, Mosnier JF, Diebold MD, Soubeyran I, Thirouard AS, Selves J, Laurent-Puig P, Ychou M. UGT1A1 polymorphism can predict hematologic toxicity in patients treated with irinotecan. Clin Cancer Res. 2007;13(11):3269-75.

15. Massacesi C, Terrazzino S, Marcucci F, Rocchi MB, Lippe P, Bisonni R, Lombardo M, Pilone A, Mattioli R, Leon A. Uridine diphosphate glucuronosyl transferase 1A1 promoter polymorphism predicts the risk of gastrointestinal toxicity and fatigue induced by irinotecan-based chemotherapy. Cancer. 2006;106(5):1007-16.

16. Marcuello E, Altés A, Menoyo A, Del Rio E, Gómez-Pardo M, Baiget M. UGT1A1 gene variations and irinotecan treatment in patients with metastatic colorectal cancer. Br J Cancer. 2004;91(4):678-82.

17. Gupta E, Lestingi TM, Mick R, Ramirez J, Vokes EE, Ratain MJ. Metabolic fate of irinotecan in humans: correlation of glucuronidation with diarrhea. Cancer Res. 1994;54(14):3723-5.
