# Supplementary figures and images for: Virtual Clinical Studies to Examine the Probability Distribution of the AUC at Target Tissues Using Physiologically-Based Pharmacokinetic Modeling: Application to Analyses of the Effect of Genetic Polymorphism of Enzymes and Transporters on Irinotecan Induced Side Effects
Source: Pharm Res. 2017 Apr 10;34(8):1584–600. doi: 10.1007/s11095-017-2153-z (PMC5498655; doi:10.1007/s11095-017-2153-z)

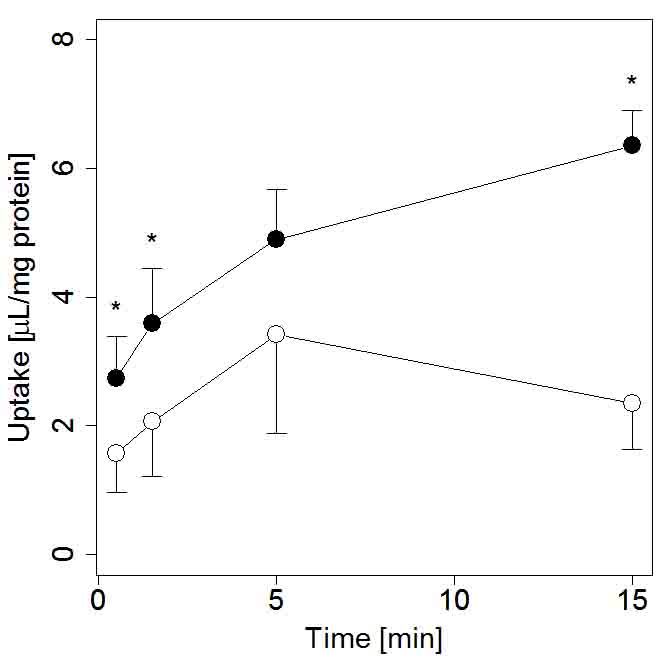

Supplement: Supplementary file 2 — Uptake of SN-38G by OATP1B1 ( N = 4). (●) and (○) and represent the uptake of SN-38G by HEK293/OATP1B1 and HEK293/mock cells, respectively. 0.3 μM SN-38G was used. * represents the p < 0.05 (Welch’s t-test) comparing the uptake amount of SN-38G between HEK293/OATP1B1 and HEK293/mock cells for each time point. The uptake of SN-38G at 15 min is 6.35 ± 0.54 (HEK293/OATP1B1) and 2.35 ± 0.71 (HEK293/mock) μL/mg protein. (JPEG 35 kb) [file 11095_2017_2153_Fig9_ESM.jpg]

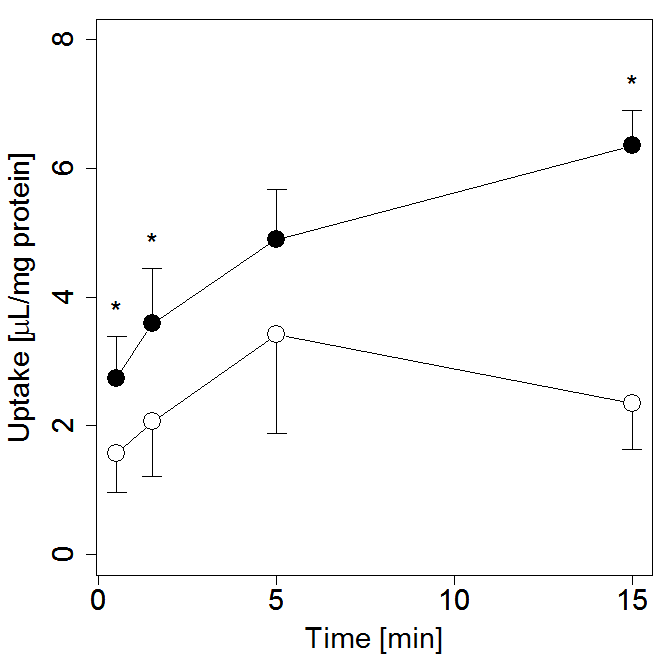

Supplement: Supplementary file 3 — High resolution image (TIFF 1321 kb) [file 11095_2017_2153_MOESM2_ESM.tif]

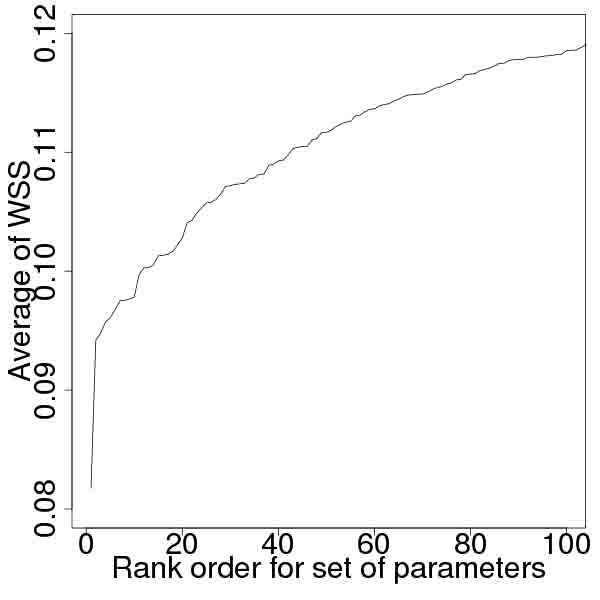

Supplement: Supplementary file 4 — Association between the average WSS and the sets of parameters obtained using CNM. The top 100 sets of parameters of 1,000,000 sets sorted in ascending order of WSS are shown. (JPEG 33 kb) [file 11095_2017_2153_Fig10_ESM.jpg]

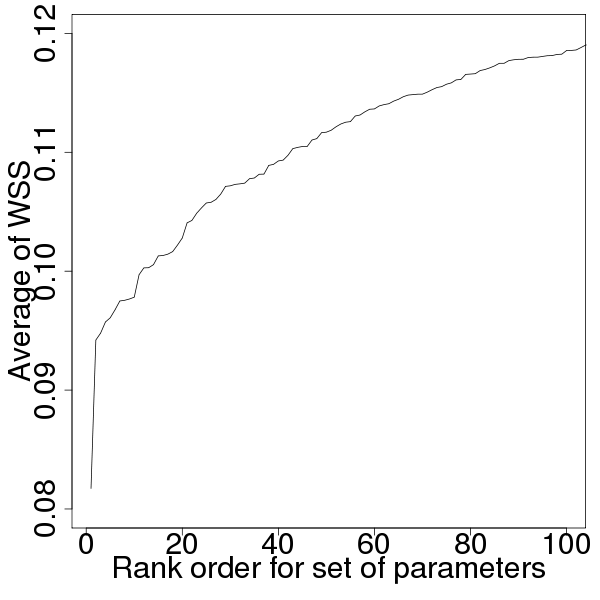

Supplement: Supplementary file 5 — High resolution image (TIFF 58 kb) [file 11095_2017_2153_MOESM3_ESM.tif]

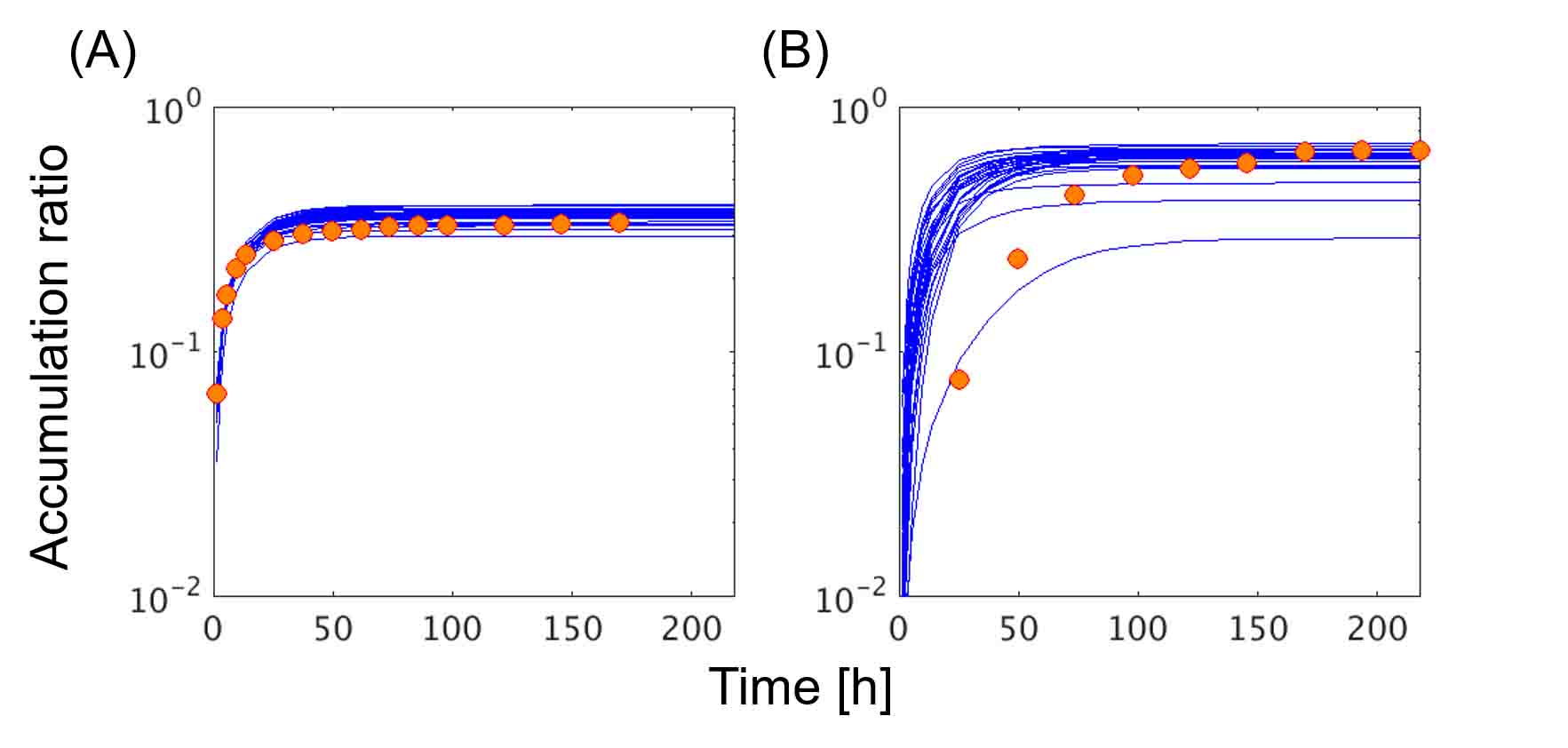

Supplement: Supplementary file 6 — Validation of the reproducibility for reported mass balance obtained in clinical study (24) using the top 30 sets of parameters. Orange dots represent the observed values that are calculated from the relative abundance of radioactive compounds in urine and feces. Each blue line represents the total amount time profile of urine (A) and feces (B). (JPEG 66 kb) [file 11095_2017_2153_Fig11_ESM.jpg]

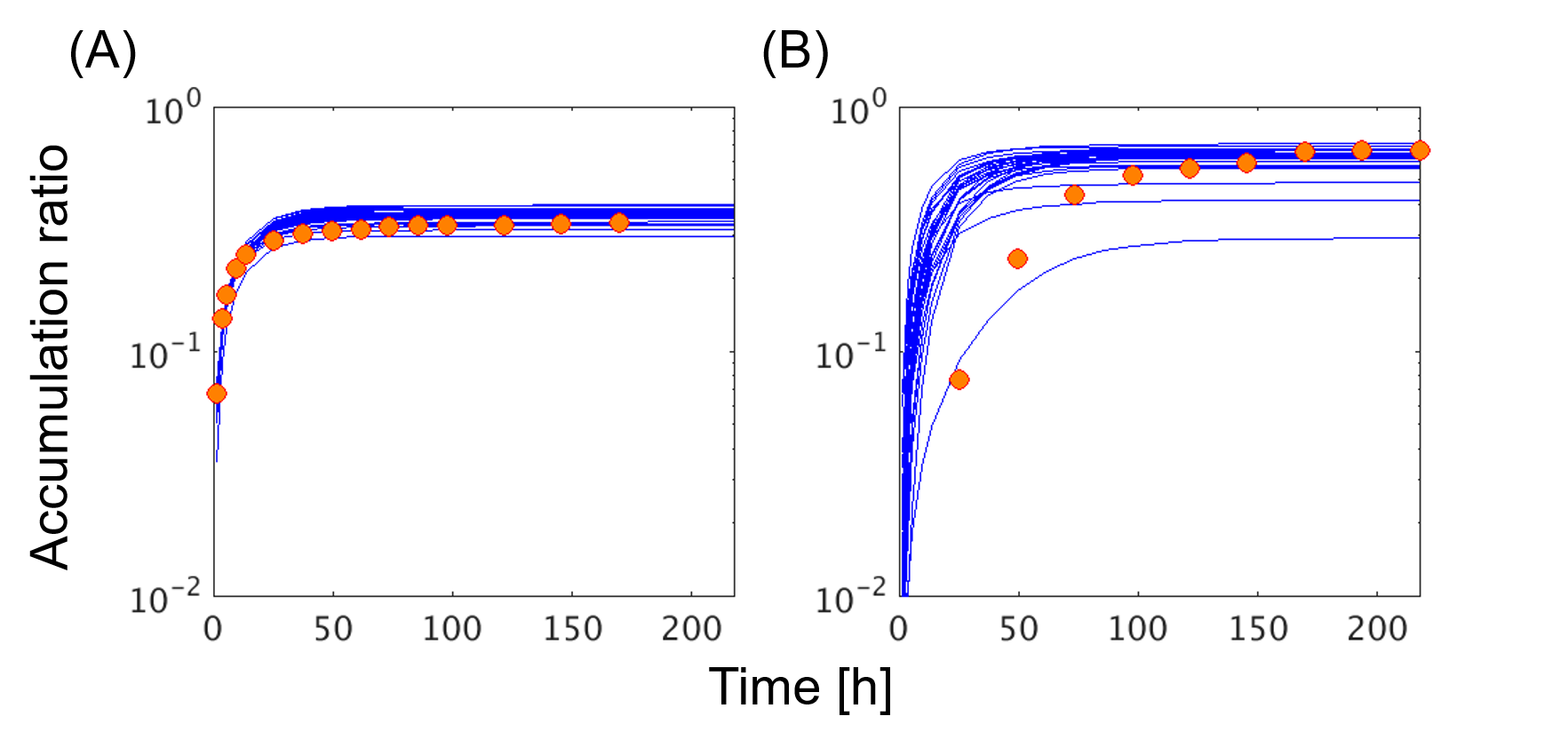

Supplement: Supplementary file 7 — High resolution image (TIFF 293 kb) [file 11095_2017_2153_MOESM4_ESM.tif]

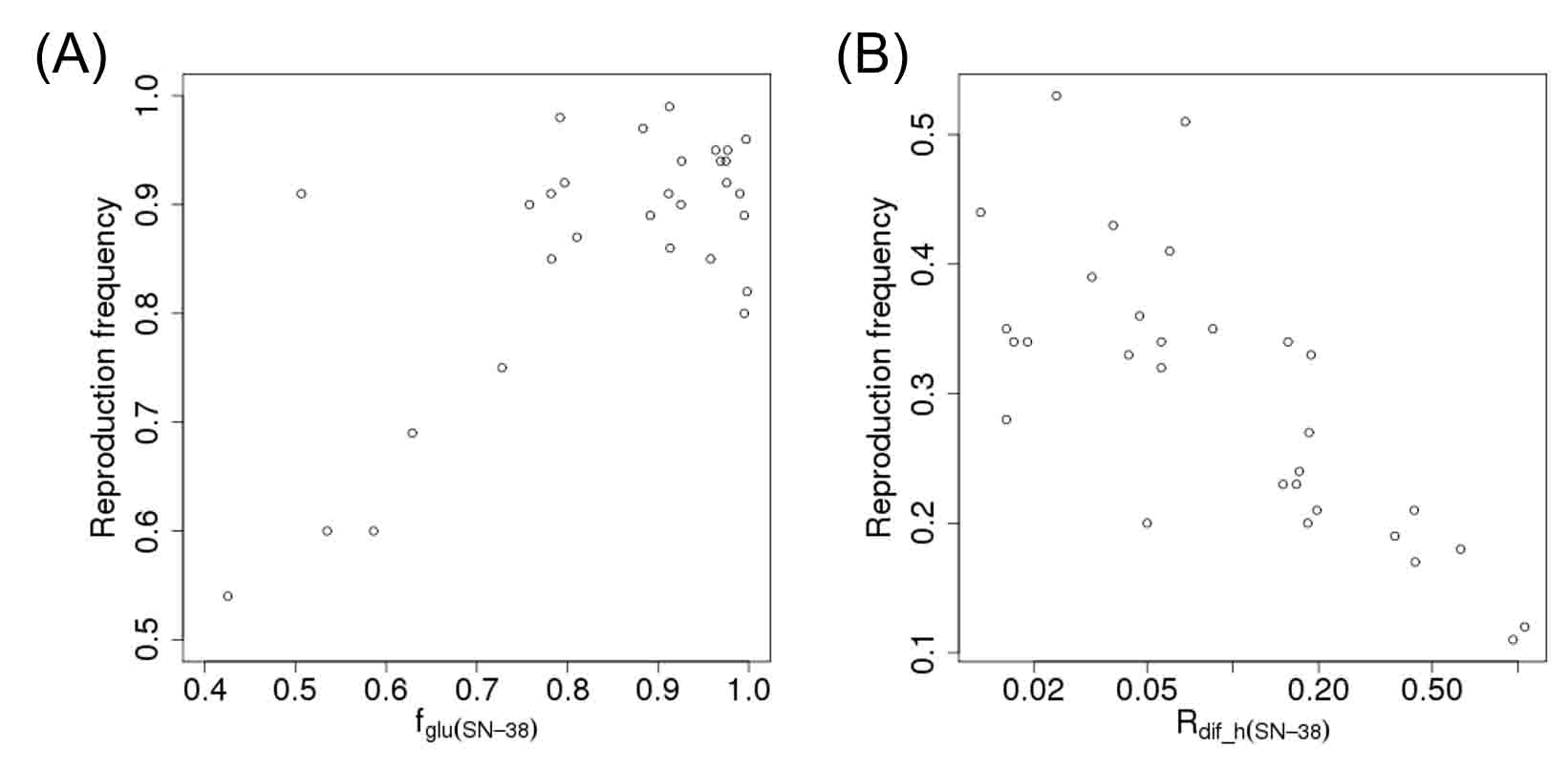

Supplement: Supplementary file 8 — Correlation among 30 sets of parameters between f glu(SN-38) (A) or R dif,h(SN-38) (B) and the frequency of reproduction of the association between neutropenia and genetic polymorphism. (A) shows the relationship between fglu(SN-38) for 30 sets of parameters and the frequency of the association between the effect of UGT1A1 *28 polymorphism and neutropenia using lager frequency cases; either dominant model (UGT1A1 *1/*1 vs. *1/*28 and *28/*28) or recessive model (UGT1A1 *1/*1 and *1/*28 vs. *28/*28) (B) shows the relationship between Rdif,h(SN-38) for 30 sets of parameters and the frequency of a significant association between the effect of SLCO1B1 c.521T>C polymorphism and neutropenia using lager frequency cases; either dominant model (SLCO1B1 521 T/T vs. T/C and C/C) or recessive model (SLCO1B1 521 T/T and T/C vs. C/C). The Rdif,h(SN-38) in X-axis of (B) is shown as a logarithmic scale. (JPEG 48 kb) [file 11095_2017_2153_Fig12_ESM.jpg]

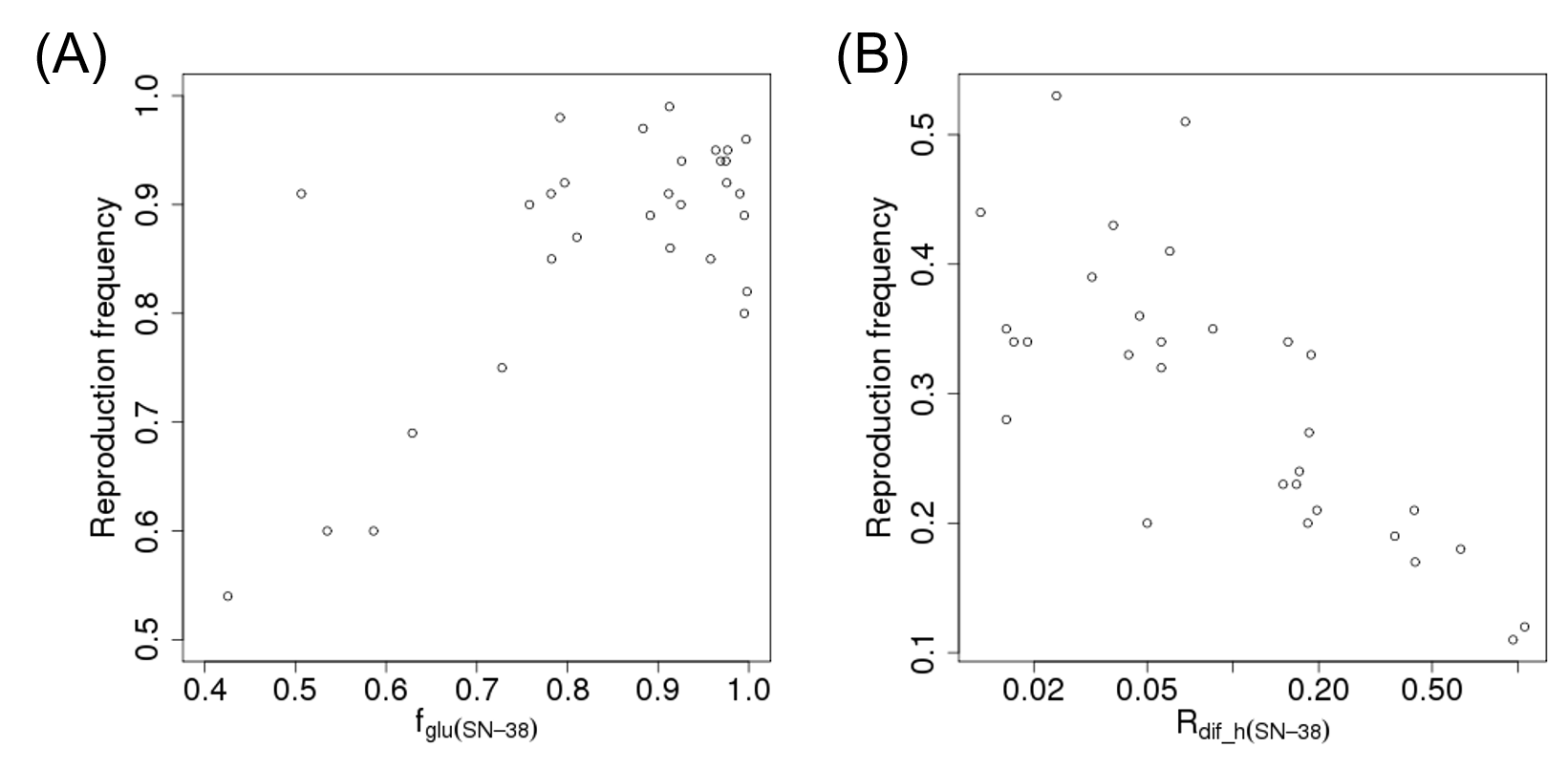

Supplement: Supplementary file 9 — High resolution image (TIFF 257 kb) [file 11095_2017_2153_MOESM5_ESM.tif]

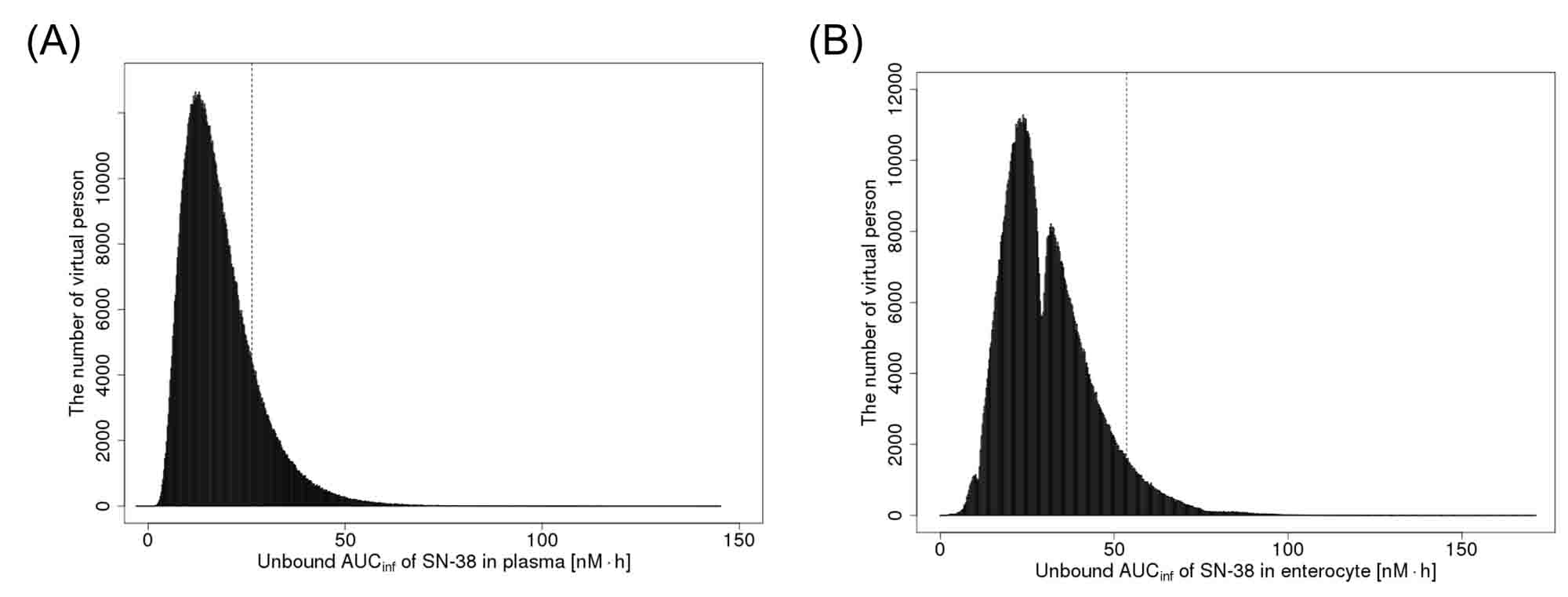

Supplement: Supplementary file 10 — Histogram of unbound plasma (A) and enterocyte (B) AUC of SN-38 among 1,000,000 virtual patients. Dotted line shows the threshold AUC for neutropenia and diarrhea (neutropenia, plasma unbound AUC > 26.35 [nM⋅h]; diarrhea, enterocyte unbound AUC > 53.60 [nM⋅h]). (JPEG 54 kb) [file 11095_2017_2153_Fig13_ESM.jpg]

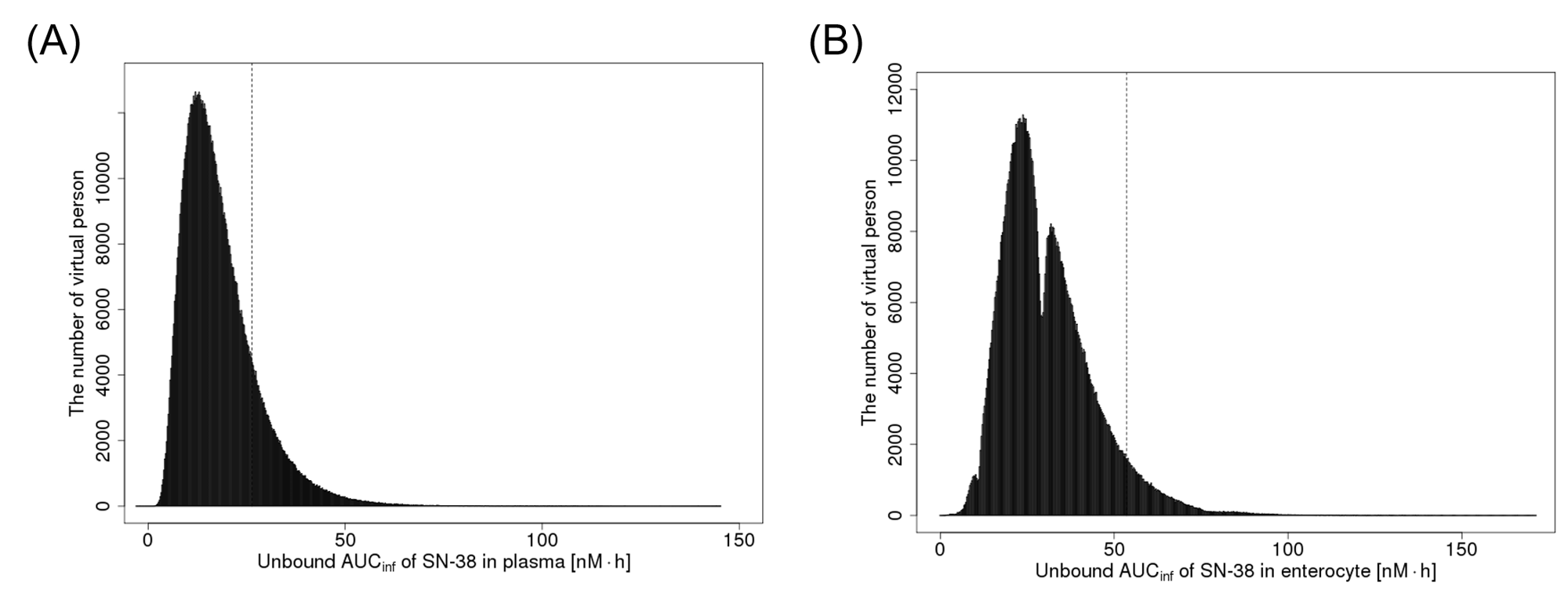

Supplement: Supplementary file 11 — High resolution image (TIFF 424 kb) [file 11095_2017_2153_MOESM6_ESM.tif]
