# Supplementary material for: Virtual Clinical Studies to Examine the Probability Distribution of the AUC at Target Tissues Using Physiologically-Based Pharmacokinetic Modeling: Application to Analyses of the Effect of Genetic Polymorphism of Enzymes and Transporters on Irinotecan Induced Side Effects
Source: Pharm Res. 2017 Apr 10;34(8):1584–600. doi: 10.1007/s11095-017-2153-z (PMC5498655; doi:10.1007/s11095-017-2153-z)
Supplement: Supplementary file 12 — (DOCX 32 kb) [file 11095_2017_2153_MOESM7_ESM.docx]

**Supplementary Table 1**

Fixed parameters used for the Cluster Newton Method

| A. Physiological parameters | | | | | | | | | | | | | | |
| --- | --- | --- | --- | --- | --- | --- | --- | --- | --- | --- | --- | --- | --- | --- |
| Parameter | | Unit | Liver | | Muscle | | Skin | | Adipose | | Mucosa (Enterocyte) | | Serosa | |
|  |  |  | Value | Reference | Value | Reference | Value | Reference | Value | Reference | Value | Reference | Value | Reference |
| Blood flow | | L/h/kg | 1.242 | (1) | 0.642 | (1) | 0.257 | (1) | 0.223 | (1) | 0.257^a^ | (1,2) | 0.274^a^ | (1, 2) |
| Volume | | L/kg | 0.0241 | (1) | 0.429 | (1) | 0.111 | (1) | 0.143 | (1) | 0.0075^a ,b^ | (3) | 0.0089^a, b^ | (3) |
| Fraction of volume | Extracellular space | - | 0.278 | (4) | - | - | - | - | - | - | 0.118 | (4) | - | - |
|  | Cells | - | 0.722 | (4) | - | - | - | - | - | - | 0.882 | (4) | - | - |
| Apical/Basolateral are ratio (AR) | | - | - | - | - | - | - | - | - | - | 20 | (5) | - | - |

| B. Pharmacokinetic parameters | | | | | | | | | | | |
| --- | --- | --- | --- | --- | --- | --- | --- | --- | --- | --- | --- |
| Parameter | Unit | Irinotecan | | SN-38 | | SN-38G | | NPC | | APC | |
|  |  | Value | Reference | Value | Reference | Value | Reference | Value | Reference | Value | Reference |
| Dose | μmol/kg | 12.66^a^ | (6) | - | - | - | - | - | - | - | - |
| CL_f_ | L/hr/kg | 0.0977^c^ | (7) | 0.0475^c^ | (7) | 0.129^c^ | (7) | 0 | Assumption  (No information) | 0.0621^c^ | (7) |
| f_p_ | - | 0.298 | *in house* | 0.0283 | *in house* | 0.691 | *in house* | 0.495 | *in house* | 0.250 | *in house* |
| f_b_ | - | 0.307 | f_p_/R_b_ | 0.022 | f_p_/R_b_ | 1.26 | f_p_/R_b_ | 0.400 | f_p_/R_b_ | 0.331 | f_p_/R_b_ |
| R_b_ | - | 0.970 | (8) | 1.31 | (8) | 0.55 | *in house* | 1.24 | *in house* | 0.755 | *in house* |
| f_h_ | - | 0.0356 | *in silico^d^* | 0.227 | *in silico^d^* | 1 | *in silico^d^* | 0.0352 | *in silico^d^* | 0.042 | *in silico^d^* |
| f_gut_ | - | 0.0563 | *in silico^d^* | 0.123 | *in silico^d^* | 1 | *in silico^d^* | 0.0614 | *in silico^d^* | 0.072 | *in silico^d^* |
| K_p,muscle_ | (tissue:blood) | 3.953 | *in silico^d^* | 0.0799 | *in silico^d^* | 0.488 | *in silico^d^* | 5.08 | *in silico^d^* | 1.48 | *in silico^d^* |
| K_p,skin_ | (tissue:blood) | 4.277 | *in silico^d^* | 0.190 | *in silico^d^* | 0.774 | *in silico^d^* | 4.25 | *in silico^d^* | 1.29 | *in silico^d^* |
| K_p,adipose_ | (tissue:blood) | 2.831 | *in silico^d^* | 0.160 | *in silico^d^* | 0.129 | *in silico^d^* | 1.23 | *in silico^d^* | 0.371 | *in silico^d^* |
| K_p,gut_ | (tissue:blood) | 6.291 | *in silico^d^* | 0.190 | *in silico^d^* | 0.671 | *in silico^d^* | 7.54 | *in silico^d^* | 2.19 | *in silico^d^* |

^a^ Calculated assuming body weight as 70 (kg).

^b^ Calculated by assuming that the drug flow from the central compartment to mucosal blood and serosa are the same.

^a^ Calculated using the weighted average value.

^d^ Calculated based on in silico estimation using clogP and pKa (9, 10).

Reference in Supplementary Table 1

1. Davies B, Morris T. Physiological parameters in laboratory animals and humans. Pharmaceutical research. 1993;10(7):1093-5.

2. Yang J, Jamei M, Yeo KR, Tucker GT, Rostami-Hodjegan A. Prediction of intestinal first-pass drug metabolism. Curr Drug Metab. 2007;8(7):676-84.

3. SimCYP version 15.

4. Kawai R, Mathew D, Tanaka C, Rowland M. Physiologically based pharmacokinetics of cyclosporine A: extension to tissue distribution kinetics in rats and scale-up to human. The Journal of pharmacology and experimental therapeutics. 1998;287(2):457-68.

5. DeSesso JM, Jacobson CF. Anatomical and physiological parameters affecting gastrointestinal absorption in humans and rats. Food Chem Toxicol 2001;39(3):209–228.

6. van der Bol JM, Loos WJ, de Jong FA, van Meerten E, Konings IR, Lam MH, de Bruijn P, Wiemer EA, Verweij J, Mathijssen RH. Effect of omeprazole on the pharmacokinetics and toxicities of irinotecan in cancer patients: a prospective cross-over drug-drug interaction study. Eur J Cancer. 2011;47(6):831-8.

7. Sai K, Kaniwa N, Itoda M, Saito Y, Hasegawa R, Komamura K, Ueno K, Kamakura S, Kitakaze M, Shirao K, Minami H, Ohtsu A, Yoshida T, Saijo N, Kitamura Y, Kamatani N, Ozawa S, Sawada J. Haplotype analysis of ABCB1/MDR1 blocks in a Japanese population reveals genotype-dependent renal clearance of irinotecan. Pharmacogenetics. 2003;13(12):741-57.

8. Combes O, Barré J, Duché JC, Vernillet L, Archimbaud Y, Marietta MP, Tillement JP, Urien S. In vitro binding and partitioning of irinotecan (CPT-11) and its metabolite, SN-38, in human blood. Invest New Drugs. 2000;18(1):1-5.

9. Rodgers T, Leahy D, Rowland M. Physiologically based pharmacokinetic modeling 1: predicting the tissue distribution of moderate-to-strong bases. J Pharm Sci. 2005;94(6):1259-76.

10. Rodgers T, Rowland M. Physiologically based pharmacokinetic modelling 2: predicting the tissue distribution of acids, very weak bases, neutrals and zwitterions. Journal of pharmaceutical sciences. 2006;95(6):1238-57.
