# Supplementary material for: Virtual Clinical Studies to Examine the Probability Distribution of the AUC at Target Tissues Using Physiologically-Based Pharmacokinetic Modeling: Application to Analyses of the Effect of Genetic Polymorphism of Enzymes and Transporters on Irinotecan Induced Side Effects
Source: Pharm Res. 2017 Apr 10;34(8):1584–600. doi: 10.1007/s11095-017-2153-z (PMC5498655; doi:10.1007/s11095-017-2153-z)
Supplement: Supplementary file 13 — (DOCX 21 kb) [file 11095_2017_2153_MOESM8_ESM.docx]

**Supplementary Table 2**

Activity ratio and allele frequency for each genetic polymorphism used for generating a virtual person.

| A. Activity ratio and allele frequency for each genetic polymorphism by generating a virtual person. | | | | | | | |
| --- | --- | --- | --- | --- | --- | --- | --- |
| Genetic polymorphism | Activity ratio to wild type (%) | | | Frequency (%) | | | |
|  | Heterozygote | Homozygote | References | Wild type | Heterozygote | Homozygote | References |
| UGT1A1 *28 | 60.2 | 32.2 | (1)^a^ | 44.66 | 47.57 | 7.77 | (2) |
| SLCO1B1 521T>C | 63.4 | 26.8 | -^b^ | See Table B | See Table B | See Table B | (3) |
| SLCO1B1 388A>G | 161 | 221 | -^b^ | See Table B | See Table B | See Table B | (3) |
| ABCG2 421C>A | 87.5 | 51.3 | -^b^ | 73.96 | 24.08 | 1.96 | (4) |
| ABCB1 3435C>T | 66.6 | 33.3 | Assumption | 23.06 | 50.30 | 26.64 | (5)^c^ |
| ABCC2 -24C>T | 66.6 | 33.3 | Assumption | 58.25 | 32.04 | 9.71 | (2) |

| B. Allele frequency between OATP1B1 521T>C and 388A>G considering linkage disequilibrium (%) | | | | |
| --- | --- | --- | --- | --- |
|  | | OATP1B1 521 | | |
|  |  | TT | TC | CC |
| SLCO1B1 388 | AA | 31.36 | 2.24 | 0.04 |
|  | AG | 29.12 | 18.96 | 0.64 |
|  | GG | 6.76 | 8.32 | 2.56 |

^a^ Activity ratio for the UGT1A1 *6 polymorphisms was used.

^b^ In-house data (unpublished)

^c^ Referred to European population values

Reference in Supplementary Table 2

1. Yamamoto K, Sato H, Fujiyama Y, Doida Y, Bamba T. Contribution of two missense mutations (G71R and Y486D) of the bilirubin UDP glycosyltransferase (UGT1A1) gene to phenotypes of Gilbert's syndrome and Crigler-Najjar syndrome type II. Biochim Biophys Acta. 1998;1406(3):267-73.

2. Teft WA, Welch S, Lenehan J, Parfitt J, Choi YH, Winquist E, Kim RB. OATP1B1 and tumour OATP1B3 modulate exposure, toxicity, and survival after irinotecan-based chemotherapy. Br J Cancer. 2015;112(5):857-65.

3. Pasanen MK, Neuvonen PJ, Niemi M. Global analysis of genetic variation in SLCO1B1. Pharmacogenomics. 2008;9(1):19-33.

4. Zamber CP, Lamba JK, Yasuda K, Farnum J, Thummel K, Schuetz JD, Schuetz EG. Natural allelic variants of breast cancer resistance protein (BCRP) and their relationship to BCRP expression in human intestine. Pharmacogenetics. 2003;13(1):19-28.

5. 1000 Genomes Project Consortium, Auton A, Brooks LD, Durbin RM, Garrison EP, Kang HM, Korbel JO, Marchini JL, McCarthy S, McVean GA, Abecasis GR. A global reference for human genetic variation. Nature. 2015;526(7571):68-74.
