# Supplementary material for: Virtual Clinical Studies to Examine the Probability Distribution of the AUC at Target Tissues Using Physiologically-Based Pharmacokinetic Modeling: Application to Analyses of the Effect of Genetic Polymorphism of Enzymes and Transporters on Irinotecan Induced Side Effects
Source: Pharm Res. 2017 Apr 10;34(8):1584–600. doi: 10.1007/s11095-017-2153-z (PMC5498655; doi:10.1007/s11095-017-2153-z)
Supplement: Supplementary file 14 — (DOCX 27 kb) [file 11095_2017_2153_MOESM9_ESM.docx]

**Supplementary Table 3**

Inter-individual variability (average, CV, and the shape of distribution) for each parameter.

| A. Physiological Parameters | | | | | | |
| --- | --- | --- | --- | --- | --- | --- |
| Parameter | Unit | Average | CV (%) | Shape of distribution | Reference of average | Reference of CV |
| V_h_ | L/kg | 0.0241 | 11.4 | Normal | (1) | (2) |
| V_muscle_ | L/kg | 0.429 | 11.4 | Normal | (1) | -^a^ |
| V_skin_ | L/kg | 0.111 | 11.4 | Normal | (1) | -^a^ |
| V_adipose_ | L/kg | 0.143 | 11.4 | Normal | (1) | -^a^ |
| V_mucosa_ | L/kg | 0.0075 | 11.4 | Normal | (3) | -^a^ |
| V_serosa_ | L/kg | 0.0089 | 11.4 | Normal | (3) | -^a^ |
| Q_h_ | L/h/kg | 1.242 | 12.2 | Normal | (1) | (2) |
| Q_muscle_ | L/h/kg | 0.642 | 12.2 | Normal | (1) | -^a^ |
| Q_skin_ | L/h/kg | 0.257 | 12.2 | Normal | (1) | -^a^ |
| Q_adipose_ | L/h/kg | 0.223 | 12.2 | Normal | (1) | -^a^ |
| Q_mucosa_ | L/h/kg | 0.257 | 12.2 | Normal | (1, 4) | -^a^ |
| Q_serosa_ | L/h/kg | 0.274 | 12.2 | Normal | (1, 4) | -^a^ |
| Fraction of parenchymal cell volume  in the whole liver | - | 0.722 | 0 | - | (5) | -^b^ |
| Fraction of epithelial cell volume  in the whole intestine | - | 0.882 | 0 | - | (5) | -^b^ |
| Apical/Basolateral are ratio (AR) | - | 20 | 0 | Normal | (6) | -^c^ |
| Filtration fraction (FF) | - | 0.199 | 14.3 | Normal | (7) | (7) |
| GFR | mL/min/1.73m^2^ | 105 | 12.7 | Normal | (7) | (7) |
| Hematocrit (Hct) | - | 0.415 | 10.5 | Normal | (8) | (8) |
| Body weight | kg | 74.87 | 15.2 | Normal | (9, 10, 11)^d^ | (9, 10, 11)^d^ |
| Body surface area (BSA) | m^2^ | - | 5.50 | Normal | (12, 13) | (12, 13) |

| B. Pharmacokinetic parameters | | | | |
| --- | --- | --- | --- | --- |
| Parameter | Unit | CV (%) | Shape of Distribution | Reference of CV |
| n[Pt] / K_d_ for albumin | - | 10.3 | Normal | (14) |
| n[Pt] / K_d_ for α1-acid glycoprotein | - | 10.3 | Normal | -^e^ |
| n[Pt] / K_d_ for red blood cells | - | 10.3 | Normal | -^e^ |
| PS_act,inf,h_ by OATP1B1 | L/h/kg | 25.8 | Lognormal | -^f^ |
| PS_dif,inf,h_ | L/h/kg | 10.0 | Lognormal | -^f,g^ |
| PS_dif,eff,h_ | L/h/kg | 10.0 | Lognormal | -^f,g^ |
| CL_met,h_ by CYP3A | L/h/kg | 33.0 | Lognormal | (14) |
| CL_met,h_ by UGT1A1 | L/h/kg | 30.0 | Lognormal | Assumption |
| CL_met,h_ by CES | L/h/kg | 30.0 | Lognormal | Assumption |
| CL_bile_ by MDR1 | L/h/kg | 25.8 | Lognormal | -^h^ |
| CL_bile_ by BCRP1 | L/h/kg | 25.8 | Lognormal | -^h^ |
| CL_bile_ by MRP2 | L/h/kg | 25.8 | Lognormal | -^h^ |
| CL_bile_ of NPC, APC | L/h/kg | 25.8 | Lognormal | -^h^ |
| k_bile_ | /h | 30.0 | Lognormal | Assumption |
| k_a_ | /h | 30.0 | Lognormal | Assumption |
| k_feces_ | /h | 30.0 | Lognormal | Assumption |
| PS_act,eff,ent_ by MDR1 | L/h/kg | 25.8 | Lognormal | -^h^ |
| PS_act,eff,ent_ by BCRP | L/h/kg | 25.8 | Lognormal | -^h^ |
| PS_act,eff,ent_ by MRP2 | L/h/kg | 25.8 | Lognormal | -^h^ |
| PS_dif,eff,ent_ | L/h/kg | 10.0 | Lognormal | -^f,g^ |
| PS_dif,inf,ent_ | L/h/kg | 10.0 | Lognormal | -^f,g^ |
| CL_met,ent_ by CYP3A | L/h/kg | 33.0 | Lognormal | (14) |
| CL_met,ent_ by UGT1A1 | L/h/kg | 30.0 | Lognormal | Assumption |
| CL_met,ent_ by CES | L/h/kg | 30.0 | Lognormal | Assumption |
| CL_int,sec_ | L/h/kg | 34.2 | Lognormal | (15) |
| K_p,muscle_ | - | - | - | -^i^ |
| K_p,skin_ | - | - | - | -^i^ |
| K_p,adipose_ | - | - | - | -^i^ |
| K_p,gut_ | - | - | - | -^i^ |
| f_t_ | - | - | - | -^i^ |
| f_gut_ | - | - | - | -^i^ |

^a^ Assuming that CV values are the same as that for the liver.

^b^ Volume fractions of parenchymal cells (liver) and epithelial cells (intestine) were assumed constant for all virtual patients.

^c^ AR value is assumed constant for all virtual patients

^d^ Determined based on the BSA distribution (See Methods section)

^e^ Assuming the same CV value as albumin concentration.

^f^ Unpublished data.

^g^ Assuming that the correlation of variance between passive diffusion of influx and efflux is 1.

^h^ Assuming the same inter-individual variability as SLCO1B1

^i^ Calculated based on in silico estimation using clogP, pKa, and the f_p_ value of each virtual person [16, 17].

Reference in Supplementary Table 3

1. Davies B, Morris T. Physiological parameters in laboratory animals and humans. Pharmaceutical research. 1993;10(7):1093-5.

2. Wynne HA, Cope LH, Mutch E, Rawlins MD, Woodhouse KW, James OF. The effect of age upon liver volume and apparent liver blood flow in healthy man. Hepatology. 1989;9(2):297-301.

3. SimCYP version 15.

4. Yang J, Jamei M, Yeo KR, Tucker GT, Rostami-Hodjegan A. Prediction of intestinal first-pass drug metabolism. Curr Drug Metab. 2007;8(7):676-84.

5. Kawai R, Mathew D, Tanaka C, Rowland M. Physiologically based pharmacokinetics of cyclosporine A: extension to tissue distribution kinetics in rats and scale-up to human. The Journal of pharmacology and experimental therapeutics. 1998;287(2):457-68.

6. DeSesso JM, Jacobson CF. Anatomical and physiological parameters affecting gastrointestinal absorption in humans and rats. Food Chem Toxicol 2001;39(3):209–228.

7. Berg UB. Differences in decline in GFR with age between males and females. Reference data on clearances of inulin and PAH in potential kidney donors. Nephrol Dial Transplant. 2006;21(9):2577-82.

8. Lin YH, Shen TY, Chang CA. Reduction of the interferences of biochemicals and hematocrit ratio on the determination of whole blood glucose using multiple screen-printed carbon electrode test strips. Anal Bioanal Chem. 2007;389(5):1623-31.

9. Myrand SP, Sekiguchi K, Man MZ, Lin X, Tzeng RY, Teng CH, Hee B, Garrett M, Kikkawa H, Lin CY, Eddy SM, Dostalik J, Mount J, Azuma J, Fujio Y, Jang IJ, Shin SG, Bleavins MR, Williams JA, Paulauskis JD, Wilner KD. Pharmacokinetics/genotype associations for major cytochrome P450 enzymes in native and first- and third-generation Japanese populations: comparison with Korean, Chinese, and Caucasian populations. Clin Pharmacol Ther. 2008;84(3):347-61.

10. Sharkey I, Boddy AV, Wallace H, Mycroft J, Hollis R, Picton S; Chemotherapy Standardisation group of the United Kingdom Children's Cancer Study Group. Body surface area estimation in children using weight alone: application in paediatric oncology. Br J Cancer. 2001;85(1):23-8.

11. Murray DM, Burmaster DE. Estimated distributions for total body surface area of men and women in the United States. J Expo Anal Environ Epidemiol. 1992;2(4):451-61.

12. van der Bol JM, Loos WJ, de Jong FA, van Meerten E, Konings IR, Lam MH, de Bruijn P, Wiemer EA, Verweij J, Mathijssen RH. Effect of omeprazole on the pharmacokinetics and toxicities of irinotecan in cancer patients: a prospective cross-over drug-drug interaction study. Eur J Cancer. 2011;47(6):831-8.

13. Crona DJ, Ramirez J, Qiao W, de Graan AJ, Ratain MJ, van Schaik RH, Mathijssen RH, Rosner GL, Innocenti F. Clinical validity of new genetic biomarkers of irinotecan neutropenia: an independent replication study. Pharmacogenomics J. 2016;16(1):54-9.

14. Kato M, Chiba K, Ito T, Koue T, Sugiyama Y. Prediction of interindividual variability in pharmacokinetics for CYP3A4 substrates in humans. Drug Metab Pharmacokinet. 2010;25(4):367-78.

15. Sai K, Kaniwa N, Itoda M, Saito Y, Hasegawa R, Komamura K, Ueno K, Kamakura S, Kitakaze M, Shirao K, Minami H, Ohtsu A, Yoshida T, Saijo N, Kitamura Y, Kamatani N, Ozawa S, Sawada J. Haplotype analysis of ABCB1/MDR1 blocks in a Japanese population reveals genotype-dependent renal clearance of irinotecan. Pharmacogenetics. 2003;13(12):741-57.

16. Rodgers T, Leahy D, Rowland M. Physiologically based pharmacokinetic modeling 1: predicting the tissue distribution of moderate-to-strong bases. J Pharm Sci. 2005;94(6):1259-76.

17. Rodgers T, Rowland M. Physiologically based pharmacokinetic modelling 2: predicting the tissue distribution of acids, very weak bases, neutrals and zwitterions. Journal of pharmaceutical sciences. 2006;95(6):1238-57.
