# Supplementary material for: Virtual Clinical Studies to Examine the Probability Distribution of the AUC at Target Tissues Using Physiologically-Based Pharmacokinetic Modeling: Application to Analyses of the Effect of Genetic Polymorphism of Enzymes and Transporters on Irinotecan Induced Side Effects
Source: Pharm Res. 2017 Apr 10;34(8):1584–600. doi: 10.1007/s11095-017-2153-z (PMC5498655; doi:10.1007/s11095-017-2153-z)
Supplement: Supplementary file 15 — (DOCX 42 kb) [file 11095_2017_2153_MOESM10_ESM.docx]

**Supplementary Table 4**

Top 30 sets of parameters determined using CNM

| A. Irinotecan parameters | | | | | | | | | | | | | | | |
| --- | --- | --- | --- | --- | --- | --- | --- | --- | --- | --- | --- | --- | --- | --- | --- |
| Parameter | | V_central_ | k_a_ | PS_dif,inf,h_ | k_bile_ | k_feces_ | CL_SN-38,h_ | CL_NPC,h_ | CL_APC,h_ | CL_others,h_ | CL_bile_ | CL_SN-38,h_ / CL_SN-38,ent_ | PS_dif,eff,ent_ | R_dif,ent_ | CL_NPC,h_ / CL_NPC,ent_ |
| Unit | | L/kg | /h | L/h/kg | /h | /h | L/h/kg | L/h/kg | L/h/kg | L/h/kg | L/h/kg | - | L/h/kg | - | - |
| Set of parameters obtained from CNM | ID 1 | 0.137 | 0.486 | 1.306 | 4.164 | 0.519 | 0.073 | 0.864 | 0.883 | 0.170 | 0.003 | 3.109 | 0.207 | 0.762 | 1.388 |
|  | ID 2 | 0.119 | 0.512 | 1.045 | 0.098 | 3.047 | 0.197 | 5.726 | 8.957 | 0.149 | 0.038 | 1.379 | 0.085 | 0.399 | 1.106 |
|  | ID 3 | 0.553 | 3.117 | 0.882 | 0.228 | 2.442 | 1.538 | 9.077 | 15.431 | 3.536 | 0.009 | 8.208 | 0.050 | 4.196 | 1.170 |
|  | ID 4 | 0.075 | 0.191 | 0.506 | 1.576 | 0.189 | 0.556 | 5.502 | 6.515 | 0.018 | 0.047 | 1.798 | 0.747 | 0.106 | 12.277 |
|  | ID 5 | 0.092 | 0.124 | 0.425 | 0.227 | 3.589 | 2.331 | 57.051 | 69.139 | 5.873 | 0.106 | 7.245 | 1.064 | 2.520 | 1.157 |
|  | ID 6 | 0.556 | 1.429 | 0.616 | 5.410 | 1.292 | 2.506 | 24.122 | 17.147 | 0.419 | 1.525 | 1.720 | 0.373 | 1.088 | 7.294 |
|  | ID 7 | 0.123 | 0.333 | 0.673 | 3.427 | 0.521 | 2.204 | 3.566 | 19.096 | 0.126 | 0.019 | 4.957 | 0.984 | 0.301 | 6.726 |
|  | ID 8 | 0.083 | 0.059 | 0.740 | 2.509 | 2.482 | 1.767 | 22.507 | 56.416 | 0.588 | 0.100 | 3.043 | 0.179 | 0.745 | 2.057 |
|  | ID 9 | 0.132 | 0.218 | 0.397 | 0.207 | 0.109 | 1.470 | 35.181 | 46.001 | 0.160 | 0.871 | 2.046 | 2.032 | 4.101 | 2.446 |
|  | ID 10 | 0.076 | 0.196 | 0.485 | 0.099 | 0.080 | 1.449 | 54.842 | 56.580 | 0.459 | 0.013 | 4.372 | 1.658 | 0.358 | 3.745 |
|  | ID 11 | 0.331 | 1.467 | 1.048 | 0.172 | 0.157 | 0.103 | 1.032 | 3.452 | 2.183 | 0.092 | 1.756 | 0.022 | 1.134 | 6.997 |
|  | ID 12 | 0.115 | 3.066 | 0.765 | 5.265 | 0.389 | 0.196 | 5.077 | 6.201 | 10.454 | 0.009 | 6.264 | 0.133 | 0.850 | 1.121 |
|  | ID 13 | 0.078 | 0.313 | 0.769 | 0.233 | 0.764 | 0.474 | 26.138 | 50.630 | 1.450 | 0.046 | 2.306 | 0.211 | 4.680 | 1.159 |
|  | ID 14 | 0.270 | 0.472 | 0.974 | 4.974 | 0.125 | 1.575 | 0.365 | 1.908 | 0.459 | 0.003 | 1.790 | 0.054 | 4.695 | 1.393 |
|  | ID 15 | 0.324 | 0.554 | 0.577 | 0.084 | 0.261 | 13.959 | 12.559 | 60.501 | 0.045 | 0.033 | 3.188 | 0.383 | 2.055 | 1.516 |
|  | ID 16 | 0.097 | 3.793 | 0.524 | 2.679 | 0.248 | 1.352 | 40.196 | 102.000 | 0.026 | 0.305 | 1.882 | 0.576 | 0.140 | 1.404 |
|  | ID 17 | 0.317 | 0.211 | 0.893 | 0.067 | 0.658 | 0.186 | 9.608 | 24.220 | 0.282 | 0.132 | 1.387 | 0.192 | 0.500 | 2.463 |
|  | ID 18 | 0.205 | 0.152 | 0.747 | 4.799 | 0.130 | 1.872 | 39.513 | 44.581 | 0.977 | 0.051 | 3.711 | 0.070 | 0.664 | 7.017 |
|  | ID 19 | 0.272 | 0.919 | 0.638 | 0.228 | 0.350 | 3.205 | 68.370 | 217.080 | 1.458 | 0.069 | 1.457 | 0.289 | 4.377 | 5.615 |
|  | ID 20 | 0.097 | 5.457 | 0.623 | 0.100 | 2.572 | 6.359 | 3.093 | 12.898 | 0.021 | 0.008 | 7.235 | 0.510 | 0.190 | 4.653 |
|  | ID 21 | 0.079 | 0.293 | 0.575 | 0.367 | 2.343 | 0.982 | 37.441 | 64.231 | 0.465 | 0.021 | 9.108 | 0.405 | 6.279 | 2.919 |
|  | ID 22 | 0.101 | 0.148 | 0.479 | 0.090 | 2.263 | 4.201 | 58.992 | 91.508 | 2.493 | 0.019 | 1.732 | 0.580 | 0.451 | 6.972 |
|  | ID 23 | 0.076 | 1.997 | 0.976 | 1.259 | 0.356 | 0.668 | 0.632 | 3.547 | 0.008 | 2.392 | 2.370 | 0.189 | 17.468 | 3.872 |
|  | ID 24 | 0.075 | 0.165 | 0.876 | 0.448 | 1.538 | 0.063 | 0.937 | 0.830 | 0.111 | 2.301 | 6.240 | 0.157 | 1.111 | 1.161 |
|  | ID 25 | 0.363 | 0.066 | 1.024 | 0.125 | 3.743 | 6.816 | 2.472 | 17.298 | 0.066 | 28.576 | 2.349 | 0.077 | 7.030 | 1.130 |
|  | ID 26 | 0.080 | 8.608 | 0.588 | 0.550 | 1.984 | 0.586 | 16.854 | 25.283 | 0.011 | 0.908 | 4.795 | 0.525 | 5.157 | 1.308 |
|  | ID 27 | 0.111 | 1.635 | 0.659 | 2.668 | 5.442 | 0.724 | 9.155 | 14.950 | 0.050 | 15.615 | 4.605 | 1.063 | 1.749 | 1.897 |
|  | ID 28 | 0.517 | 0.277 | 0.600 | 0.354 | 0.509 | 1.442 | 38.355 | 32.022 | 0.042 | 11.461 | 2.357 | 0.424 | 0.128 | 5.560 |
|  | ID 29 | 1.139 | 4.522 | 0.704 | 1.145 | 1.157 | 0.311 | 1.738 | 3.487 | 0.024 | 0.006 | 1.392 | 1.875 | 0.050 | 1.718 |
|  | ID 30 | 0.075 | 2.901 | 0.398 | 3.878 | 0.054 | 0.872 | 12.242 | 18.722 | 7.098 | 0.008 | 3.170 | 0.685 | 1.432 | 2.339 |
| CV (%) calculated by  30 sets | | 103 | 137 | 31.3 | 118 | 105 | 141 | 103 | 121 | 187 | 281 | 62.9 | 103 | 140 | 81.5 |

| B. SN-38 parameters | | | | | | | | | | | |
| --- | --- | --- | --- | --- | --- | --- | --- | --- | --- | --- | --- |
| Parameter | | V_central_ | k_a_ | R_dif,h_ | 1/β | CL_int,all_ | 1/f_glu_^a^ | k_bile_ | k_feces_ | R_dif,ent_ | CL_SN-38G,h_ / CL_SN-38G,ent_ |
| Unit | | L/kg | /h | - | - | L/h/kg | - | /h | /h | - | - |
| Set of parameters obtained from CNM | ID 1 | 0.108 | 1.670 | 0.056 | 1.310 | 8.647 | 1.038 | 0.101 | 0.103 | 1.301 | 1.167 |
|  | ID 2 | 0.078 | 6.454 | 0.188 | 1.388 | 12.078 | 1.319 | 0.053 | 1.053 | 7.277 | 1.852 |
|  | ID 3 | 0.212 | 2.379 | 0.060 | 1.869 | 29.941 | 1.234 | 0.323 | 0.251 | 0.411 | 2.209 |
|  | ID 4 | 0.076 | 8.358 | 0.167 | 1.492 | 13.050 | 1.374 | 0.787 | 0.229 | 2.857 | 1.899 |
|  | ID 5 | 0.125 | 5.371 | 0.068 | 1.663 | 27.793 | 2.350 | 0.111 | 0.144 | 11.554 | 1.265 |
|  | ID 6 | 0.084 | 1.206 | 0.156 | 1.506 | 21.852 | 1.706 | 0.390 | 0.144 | 3.621 | 4.042 |
|  | ID 7 | 0.082 | 1.397 | 0.032 | 1.799 | 26.446 | 1.095 | 0.829 | 0.051 | 1.199 | 2.309 |
|  | ID 8 | 0.093 | 1.323 | 1.057 | 1.223 | 5.655 | 1.279 | 4.999 | 0.080 | 1.156 | 2.312 |
|  | ID 9 | 0.130 | 1.576 | 0.019 | 1.362 | 9.359 | 1.002 | 0.152 | 0.032 | 0.344 | 1.478 |
|  | ID 10 | 0.105 | 7.695 | 0.085 | 1.853 | 18.893 | 1.005 | 0.166 | 0.090 | 4.501 | 7.398 |
|  | ID 11 | 0.079 | 0.936 | 0.016 | 1.326 | 6.892 | 1.024 | 0.162 | 0.108 | 0.613 | 2.687 |
|  | ID 12 | 0.257 | 4.313 | 0.017 | 1.573 | 15.532 | 1.097 | 1.515 | 0.071 | 0.266 | 3.188 |
|  | ID 13 | 0.077 | 1.684 | 0.050 | 1.139 | 2.125 | 1.081 | 0.784 | 0.390 | 1.999 | 3.119 |
|  | ID 14 | 0.088 | 4.013 | 0.038 | 1.330 | 48.131 | 1.080 | 2.434 | 0.061 | 1.884 | 1.942 |
|  | ID 15 | 0.077 | 0.377 | 0.171 | 1.135 | 5.915 | 1.590 | 2.553 | 0.082 | 4.641 | 1.911 |
|  | ID 16 | 0.080 | 1.077 | 0.047 | 1.745 | 21.426 | 1.122 | 0.135 | 0.060 | 1.855 | 6.751 |
|  | ID 17 | 0.101 | 5.115 | 0.056 | 1.928 | 9.509 | 1.044 | 0.373 | 0.050 | 0.430 | 5.086 |
|  | ID 18 | 0.081 | 1.866 | 0.013 | 1.354 | 17.657 | 1.278 | 2.168 | 0.138 | 1.793 | 1.840 |
|  | ID 19 | 0.077 | 4.878 | 0.436 | 1.557 | 9.315 | 1.032 | 3.949 | 0.182 | 0.634 | 2.131 |
|  | ID 20 | 0.524 | 2.694 | 0.185 | 1.260 | 16.074 | 1.025 | 1.129 | 0.274 | 8.774 | 1.465 |
|  | ID 21 | 0.076 | 0.744 | 0.150 | 1.235 | 6.153 | 1.026 | 2.425 | 0.058 | 8.403 | 1.093 |
|  | ID 22 | 0.076 | 7.737 | 0.043 | 1.951 | 17.175 | 1.005 | 3.051 | 0.170 | 1.902 | 1.195 |
|  | ID 23 | 0.078 | 2.426 | 0.962 | 1.698 | 16.930 | 1.096 | 3.069 | 0.047 | 1.000 | 1.507 |
|  | ID 24 | 0.105 | 5.302 | 0.433 | 1.222 | 8.389 | 1.974 | 0.185 | 0.069 | 0.250 | 2.101 |
|  | ID 25 | 0.117 | 3.177 | 0.370 | 1.139 | 6.056 | 1.255 | 0.075 | 0.196 | 0.364 | 2.072 |
|  | ID 26 | 0.080 | 7.279 | 0.183 | 1.137 | 2.945 | 1.132 | 0.140 | 0.863 | 0.774 | 1.120 |
|  | ID 27 | 0.147 | 2.746 | 0.630 | 2.312 | 17.164 | 1.003 | 0.512 | 0.048 | 3.959 | 8.407 |
|  | ID 28 | 0.080 | 4.725 | 0.197 | 1.063 | 4.074 | 1.263 | 0.051 | 0.166 | 1.465 | 1.331 |
|  | ID 29 | 0.095 | 2.944 | 0.024 | 2.564 | 54.457 | 1.869 | 0.867 | 0.128 | 5.967 | 2.818 |
|  | ID 30 | 0.093 | 5.193 | 0.016 | 2.107 | 17.006 | 1.010 | 3.397 | 0.179 | 0.873 | 10.435 |
| CV (%) calculated by  30 sets | | 75.3 | 66.0 | 135 | 24.5 | 76.6 | 26.7 | 113 | 123 | 108 | 80.0 |

| C. SN-38G parameters | | | | | | | | | | |
| --- | --- | --- | --- | --- | --- | --- | --- | --- | --- | --- |
| Parameter | | V_central_ | k_a_ | R_dif,h_ | 1/β | CL_int,all_ | k_bile_ | k_feces_ | R_dif,ent_ | k_dec_ |
| Unit | | L/kg | /h | - | - | L/h/kg | /h | /h | - | /h |
| Set of parameters obtained from CNM | ID 1 | 0.094 | 5.418 | 0.401 | 3.098 | 0.137 | 0.675 | 0.100 | 5.025 | 1.137 |
|  | ID 2 | 0.289 | 1.527 | 0.924 | 9.950 | 0.156 | 0.156 | 0.224 | 3.103 | 0.083 |
|  | ID 3 | 0.525 | 1.158 | 0.101 | 11.643 | 0.333 | 0.128 | 0.469 | 0.269 | 3.987 |
|  | ID 4 | 0.088 | 0.587 | 0.106 | 5.065 | 0.049 | 0.216 | 0.103 | 3.540 | 1.332 |
|  | ID 5 | 0.080 | 0.710 | 0.287 | 5.430 | 0.052 | 0.193 | 0.276 | 2.340 | 0.296 |
|  | ID 6 | 0.156 | 0.289 | 0.253 | 4.903 | 0.102 | 0.171 | 0.068 | 2.084 | 0.805 |
|  | ID 7 | 0.080 | 0.157 | 0.215 | 8.661 | 0.112 | 0.133 | 0.140 | 5.203 | 1.170 |
|  | ID 8 | 0.170 | 1.105 | 0.095 | 59.404 | 0.053 | 0.683 | 0.082 | 2.035 | 2.278 |
|  | ID 9 | 0.218 | 0.201 | 0.144 | 13.322 | 0.038 | 0.064 | 0.126 | 4.944 | 2.454 |
|  | ID 10 | 0.120 | 1.949 | 0.101 | 13.664 | 0.048 | 0.064 | 0.082 | 2.241 | 5.441 |
|  | ID 11 | 0.075 | 2.218 | 0.052 | 8.619 | 0.119 | 0.077 | 0.953 | 2.012 | 0.772 |
|  | ID 12 | 0.130 | 1.168 | 0.368 | 2.081 | 0.074 | 0.084 | 2.485 | 0.814 | 4.227 |
|  | ID 13 | 0.078 | 3.937 | 0.055 | 81.354 | 0.019 | 0.129 | 1.276 | 0.364 | 3.490 |
|  | ID 14 | 0.145 | 1.191 | 0.114 | 3.544 | 1.204 | 0.533 | 0.096 | 4.369 | 1.340 |
|  | ID 15 | 0.126 | 0.166 | 0.026 | 25.684 | 0.160 | 0.161 | 1.046 | 7.150 | 2.695 |
|  | ID 16 | 0.086 | 0.099 | 0.118 | 72.550 | 0.058 | 0.346 | 0.546 | 0.127 | 0.982 |
|  | ID 17 | 0.075 | 0.337 | 0.433 | 29.369 | 0.023 | 0.646 | 0.189 | 1.307 | 1.154 |
|  | ID 18 | 0.176 | 2.096 | 0.014 | 10.915 | 0.312 | 0.183 | 0.073 | 0.325 | 1.123 |
|  | ID 19 | 0.087 | 0.754 | 0.077 | 18.298 | 0.046 | 0.199 | 0.083 | 4.437 | 4.242 |
|  | ID 20 | 0.437 | 3.852 | 0.017 | 6.327 | 0.574 | 0.212 | 0.501 | 0.428 | 1.710 |
|  | ID 21 | 0.166 | 2.207 | 0.038 | 27.811 | 0.064 | 0.061 | 0.167 | 0.140 | 1.231 |
|  | ID 22 | 0.075 | 0.676 | 0.046 | 2.807 | 0.054 | 0.433 | 0.179 | 0.475 | 1.575 |
|  | ID 23 | 0.249 | 0.144 | 0.044 | 169.170 | 0.092 | 1.393 | 0.174 | 0.442 | 0.677 |
|  | ID 24 | 0.081 | 2.869 | 0.110 | 6.938 | 0.121 | 1.012 | 0.784 | 4.276 | 0.446 |
|  | ID 25 | 0.243 | 2.104 | 0.048 | 80.881 | 0.237 | 0.065 | 0.096 | 6.595 | 0.881 |
|  | ID 26 | 0.184 | 1.162 | 0.017 | 49.264 | 0.074 | 0.081 | 0.561 | 1.093 | 0.201 |
|  | ID 27 | 0.078 | 1.208 | 0.348 | 25.970 | 0.050 | 0.102 | 0.177 | 0.214 | 4.151 |
|  | ID 28 | 0.078 | 0.069 | 0.357 | 2.959 | 0.164 | 0.139 | 2.723 | 0.208 | 1.156 |
|  | ID 29 | 0.076 | 0.188 | 0.190 | 2.286 | 0.060 | 0.112 | 0.175 | 7.820 | 1.444 |
|  | ID 30 | 0.077 | 9.488 | 0.160 | 12.082 | 0.068 | 0.050 | 0.121 | 3.482 | 0.129 |
| CV (%) calculated by  30 sets | | 71.8 | 121 | 108 | 139 | 148 | 116 | 141 | 90.1 | 82.0 |

| D. NPC parameters | | | | | | | | |
| --- | --- | --- | --- | --- | --- | --- | --- | --- |
| Parameter | | V_central_ | k_a_ | 1/β | CL_int,all_ | 1/f_bile_ | k_bile_ | k_feces_ |
| Unit | | L/kg | /h | - | L/h/kg | - | /h | /h |
| Set of parameters obtained from CNM | ID 1 | 0.651 | 0.626 | 2.267 | 10.573 | 1.321 | 0.897 | 0.224 |
|  | ID 2 | 0.147 | 2.147 | 3.631 | 13.652 | 1.441 | 0.169 | 0.045 |
|  | ID 3 | 0.077 | 0.064 | 3.665 | 11.155 | 2.268 | 2.506 | 1.075 |
|  | ID 4 | 0.099 | 0.100 | 1.270 | 2.062 | 1.436 | 0.076 | 0.061 |
|  | ID 5 | 0.259 | 1.127 | 1.588 | 3.711 | 23.521 | 0.320 | 1.547 |
|  | ID 6 | 0.087 | 1.388 | 2.882 | 9.704 | 1.522 | 0.196 | 3.884 |
|  | ID 7 | 0.075 | 0.109 | 30.205 | 3.736 | 8.018 | 2.175 | 0.246 |
|  | ID 8 | 0.075 | 0.117 | 1.405 | 3.398 | 1.443 | 0.198 | 0.422 |
|  | ID 9 | 0.109 | 0.679 | 1.873 | 3.263 | 2.565 | 0.150 | 0.943 |
|  | ID 10 | 0.079 | 2.642 | 2.314 | 7.874 | 1.702 | 0.235 | 0.562 |
|  | ID 11 | 0.109 | 0.348 | 7.251 | 5.368 | 2.588 | 0.132 | 0.085 |
|  | ID 12 | 0.688 | 0.049 | 3.465 | 7.652 | 3.047 | 0.062 | 1.068 |
|  | ID 13 | 0.430 | 1.077 | 2.265 | 6.169 | 1.255 | 0.073 | 0.069 |
|  | ID 14 | 0.747 | 0.254 | 28.768 | 4.063 | 2.798 | 0.657 | 0.857 |
|  | ID 15 | 0.075 | 0.071 | 17.540 | 3.876 | 1.967 | 0.760 | 0.590 |
|  | ID 16 | 0.132 | 0.837 | 1.610 | 2.338 | 4.568 | 7.644 | 0.562 |
|  | ID 17 | 0.111 | 0.222 | 1.816 | 4.064 | 1.303 | 2.362 | 0.094 |
|  | ID 18 | 0.099 | 0.066 | 1.620 | 9.847 | 1.730 | 1.107 | 2.890 |
|  | ID 19 | 0.749 | 0.217 | 3.641 | 6.768 | 1.590 | 0.840 | 0.068 |
|  | ID 20 | 0.076 | 3.026 | 12.786 | 3.991 | 1.833 | 0.111 | 0.084 |
|  | ID 21 | 0.077 | 0.269 | 1.789 | 5.955 | 1.566 | 0.119 | 0.290 |
|  | ID 22 | 0.591 | 0.804 | 3.897 | 8.142 | 1.550 | 3.900 | 1.519 |
|  | ID 23 | 0.076 | 1.029 | 9.125 | 2.712 | 1.628 | 0.090 | 0.029 |
|  | ID 24 | 0.084 | 3.408 | 1.586 | 3.226 | 6.765 | 1.944 | 0.578 |
|  | ID 25 | 0.540 | 2.749 | 16.961 | 1.747 | 1.941 | 0.501 | 2.564 |
|  | ID 26 | 0.767 | 1.638 | 1.756 | 5.113 | 1.993 | 0.186 | 1.899 |
|  | ID 27 | 0.320 | 1.102 | 4.253 | 4.714 | 5.886 | 1.169 | 1.993 |
|  | ID 28 | 0.223 | 0.191 | 1.441 | 5.214 | 3.796 | 0.094 | 0.185 |
|  | ID 29 | 0.334 | 2.092 | 7.826 | 7.519 | 16.910 | 0.056 | 0.073 |
|  | ID 30 | 0.077 | 3.145 | 1.946 | 2.975 | 2.613 | 0.776 | 1.920 |
| CV (%) calculated by  30 sets | | 94.1 | 101 | 127 | 53.1 | 129 | 159 | 112 |

| E. APC parameters | | | | | | | |
| --- | --- | --- | --- | --- | --- | --- | --- |
| Parameter | | V_central_ | k_a_ | 1/β | CL_int,all_ | k_bile_ | k_feces_ |
| Unit | | L/kg | /h | - | L/h/kg | /h | /h |
| Set of parameters obtained from CNM | ID 1 | 0.075 | 3.895 | 41.336 | 0.379 | 0.549 | 2.647 |
|  | ID 2 | 0.087 | 0.253 | 16.932 | 1.428 | 4.031 | 2.236 |
|  | ID 3 | 0.078 | 1.272 | 52.442 | 1.267 | 0.431 | 0.177 |
|  | ID 4 | 0.075 | 0.403 | 6.428 | 0.120 | 1.917 | 0.915 |
|  | ID 5 | 0.076 | 4.005 | 50.411 | 0.152 | 3.420 | 2.955 |
|  | ID 6 | 0.075 | 0.365 | 66.130 | 0.068 | 0.140 | 0.117 |
|  | ID 7 | 0.557 | 3.170 | 26.321 | 0.448 | 2.629 | 0.453 |
|  | ID 8 | 0.637 | 0.645 | 32.153 | 0.806 | 0.084 | 0.446 |
|  | ID 9 | 0.192 | 2.047 | 15.117 | 0.144 | 0.203 | 0.092 |
|  | ID 10 | 0.079 | 4.272 | 8.727 | 0.067 | 1.059 | 0.790 |
|  | ID 11 | 0.114 | 0.390 | 4.769 | 0.962 | 2.155 | 2.558 |
|  | ID 12 | 0.075 | 0.742 | 4.506 | 0.278 | 0.083 | 0.129 |
|  | ID 13 | 0.109 | 0.463 | 2.702 | 0.715 | 0.197 | 1.394 |
|  | ID 14 | 0.084 | 0.389 | 27.382 | 0.759 | 0.455 | 0.148 |
|  | ID 15 | 0.094 | 0.190 | 3.392 | 0.542 | 0.571 | 0.073 |
|  | ID 16 | 0.076 | 0.887 | 39.100 | 0.419 | 0.917 | 0.899 |
|  | ID 17 | 0.489 | 1.006 | 16.655 | 0.933 | 0.343 | 0.892 |
|  | ID 18 | 0.309 | 0.179 | 24.545 | 0.495 | 0.266 | 0.518 |
|  | ID 19 | 0.143 | 1.137 | 4.803 | 1.167 | 2.390 | 1.523 |
|  | ID 20 | 0.096 | 1.641 | 90.655 | 0.407 | 11.769 | 0.028 |
|  | ID 21 | 0.076 | 3.599 | 5.336 | 0.510 | 0.728 | 4.649 |
|  | ID 22 | 0.103 | 4.621 | 13.277 | 0.308 | 0.379 | 0.353 |
|  | ID 23 | 0.075 | 1.227 | 49.044 | 0.684 | 0.456 | 0.239 |
|  | ID 24 | 0.077 | 9.647 | 54.894 | 0.064 | 1.709 | 2.841 |
|  | ID 25 | 0.080 | 2.831 | 10.185 | 0.512 | 2.795 | 0.321 |
|  | ID 26 | 0.075 | 0.231 | 6.342 | 0.298 | 3.506 | 0.121 |
|  | ID 27 | 0.677 | 3.755 | 19.710 | 0.119 | 3.998 | 0.472 |
|  | ID 28 | 0.077 | 0.646 | 116.990 | 0.102 | 0.366 | 0.126 |
|  | ID 29 | 0.097 | 4.207 | 354.470 | 0.126 | 3.866 | 0.510 |
|  | ID 30 | 0.081 | 0.522 | 19.549 | 0.105 | 0.293 | 0.647 |
| CV (%) calculated by  30 sets | | 108 | 107 | 166 | 79.7 | 135 | 117 |

^a^ obtained by the following equation, f_glu_ = 1 - f_bile_
