# Supplementary material for: Virtual Clinical Studies to Examine the Probability Distribution of the AUC at Target Tissues Using Physiologically-Based Pharmacokinetic Modeling: Application to Analyses of the Effect of Genetic Polymorphism of Enzymes and Transporters on Irinotecan Induced Side Effects
Source: Pharm Res. 2017 Apr 10;34(8):1584–600. doi: 10.1007/s11095-017-2153-z (PMC5498655; doi:10.1007/s11095-017-2153-z)
Supplement: Supplementary file 17 — (DOCX 20 kb) [file 11095_2017_2153_MOESM12_ESM.docx]

**Supplementary Table 6**

Frequency of neutropenia and diarrhea reported in clinical studies.

| Reference | [1] | [2] | | [3] | [4] | [5] | [6] | | Average±SD |
| --- | --- | --- | --- | --- | --- | --- | --- | --- | --- |
| Treatment | FOLFIRI^a^ | FOLFIRI^a^ | FOLFIRI^a^ | Irinotecan | FOLFIRI^a^ | FOLFIRI^a^ | FOLFIRI^a^ | FOLFIRI^a^ | - |
| The number of patient | 602 | 294 | 246 | 629 | 536 | 605 | 110 | 82 | 388±231 |
| The number of grade  3 or 4 neutropenia | 148  (24.6%) | 68  (23.1%) | 43  (17.5%) | 151  (24.0%) | 123  (22.9%) | (29.5%)^b^ | 24  (21.8%) | 17  (20.7%) | 23.0±3.4 (%) |
| The number of grade  3 or 4 diarrhea | 63  (10.5%) | 27  (9.2%) | 26  (10.6%) | 99  (15.7%) | 51  (9.5%) | (7.8%)^b^ | 14  (12.7%) | 5  (6.1%) | 10.3±3.0 (%) |

^a^ FOLFIRI; fluorouracil, leucovorin, and irinotecan

^b^ Described only as percentage.

Reference in Supplementary Table 6

1. Van Cutsem E, Köhne CH, Hitre E, Zaluski J, Chang Chien CR, Makhson A, D'Haens G, Pintér T, Lim R, Bodoky G, Roh JK, Folprecht G, Ruff P, Stroh C, Tejpar S, Schlichting M, Nippgen J, Rougier P. Cetuximab and chemotherapy as initial treatment for metastatic colorectal cancer. N Engl J Med. 2009;360(14):1408-17.

2. Peeters M, Price TJ, Cervantes A, Sobrero AF, Ducreux M, Hotko Y, André T, Chan E, Lordick F, Punt CJ, Strickland AH, Wilson G, Ciuleanu TE, Roman L, Van Cutsem E, Tzekova V, Collins S, Oliner KS, Rong A, Gansert J. Randomized phase III study of panitumumab with fluorouracil, leucovorin, and irinotecan (FOLFIRI) compared with FOLFIRI alone as second-line treatment in patients with metastatic colorectal cancer. J Clin Oncol. 2010;28(31):4706-13.

3. Sobrero AF, Maurel J, Fehrenbacher L, Scheithauer W, Abubakr YA, Lutz MP, Vega-Villegas ME, Eng C, Steinhauer EU, Prausova J, Lenz HJ, Borg C, Middleton G, Kröning H, Luppi G, Kisker O, Zubel A, Langer C, Kopit J, Burris HA 3rd. EPIC: phase III trial of cetuximab plus irinotecan after fluoropyrimidine and oxaliplatin failure in patients with metastatic colorectal cancer. J Clin Oncol. 2008;26(14):2311-9.

4. Tabernero J, Yoshino T, Cohn AL, Obermannova R, Bodoky G, Garcia-Carbonero R, Ciuleanu TE, Portnoy DC, Van Cutsem E, Grothey A, Prausová J, Garcia-Alfonso P, Yamazaki K, Clingan PR, Lonardi S, Kim TW, Simms L, Chang SC, Nasroulah F; RAISE Study Investigators.. Ramucirumab versus placebo in combination with second-line FOLFIRI in patients with metastatic colorectal carcinoma that progressed during or after first-line therapy with bevacizumab, oxaliplatin, and a fluoropyrimidine (RAISE): a randomised, double-blind, multicentre, phase 3 study. Lancet Oncol. 2015;16(5):499-508.

5. Van Cutsem E, Tabernero J, Lakomy R, Prenen H, Prausová J, Macarulla T, Ruff P, van Hazel GA, Moiseyenko V, Ferry D, McKendrick J, Polikoff J, Tellier A, Castan R, Allegra C. Addition of aflibercept to fluorouracil, leucovorin, and irinotecan improves survival in a phase III randomized trial in patients with metastatic colorectal cancer previously treated with an oxaliplatin-based regimen. J Clin Oncol. 2012;30(28):3499-506.

6. Tournigand C, André T, Achille E, Lledo G, Flesh M, Mery-Mignard D, Quinaux E, Couteau C, Buyse M, Ganem G, Landi B, Colin P, Louvet C, de Gramont A. FOLFIRI followed by FOLFOX6 or the reverse sequence in advanced colorectal cancer: a randomized GERCOR study. J Clin Oncol. 2004;22(2):229-37.
